# A synthetic promoter system for well-controlled protein expression with different carbon sources in *Saccharomyces cerevisiae*

**DOI:** 10.1186/s12934-021-01691-3

**Published:** 2021-10-18

**Authors:** Jiliang Deng, Yanling Wu, Zhaohui Zheng, Nanzhu Chen, Xiaozhou Luo, Hongting Tang, Jay D. Keasling

**Affiliations:** 1 Center for Synthetic Biochemistry, Shenzhen Institute of Synthetic Biology, Shenzhen Institute for Advanced Technology, Chinese Academy of Sciences, Shenzhen, 518055 China; 2 Joint BioEnergy Institute, Emeryville, CA 94608 USA; 3 Biological Systems and Engineering Division, Lawrence Berkeley National Laboratory, Berkeley, CA 94720 USA; 4 Department of Chemical and Biomolecular Engineering & Department of Bioengineering, University of California, Berkeley, CA 94720 USA; 5 Novo Nordisk Foundation Center for Biosustainability, Technical University of Denmark, 2800 Kgs. Lyngby, Denmark; 6 Present Address: State Key Laboratory of Hybrid Rice, Department of Plant Science, College of Life Sciences, Wuhan University, Wuhan, 430072 China

**Keywords:** Synthetic promoter, Carbon sources, Protein expression, *Saccharomyces cerevisiae*

## Abstract

**Background:**

*Saccharomyces cerevisiae* is an important synthetic biology chassis for microbial production of valuable molecules. Promoter engineering has been frequently applied to generate more synthetic promoters with a variety of defined characteristics in order to achieve a well-regulated genetic network for high production efficiency. Galactose-inducible (GAL) expression systems, composed of GAL promoters and multiple GAL regulators, have been widely used for protein overexpression and pathway construction in *S. cerevisiae*. However, the function of each element in synthetic promoters and how they interact with GAL regulators are not well known.

**Results:**

Here, a library of synthetic GAL promoters demonstrate that upstream activating sequences (UASs) and core promoters have a synergistic relationship that determines the performance of each promoter under different carbon sources. We found that the strengths of synthetic GAL promoters could be fine-tuned by manipulating the sequence, number, and substitution of UASs. Core promoter replacement generated synthetic promoters with a twofold strength improvement compared with the GAL1 promoter under multiple different carbon sources in a strain with GAL1 and GAL80 engineering. These results represent an expansion of the classic GAL expression system with an increased dynamic range and a good tolerance of different carbon sources.

**Conclusions:**

In this study, the effect of each element on synthetic GAL promoters has been evaluated and a series of well-controlled synthetic promoters are constructed. By studying the interaction of synthetic promoters and GAL regulators, synthetic promoters with an increased dynamic range under different carbon sources are created.

**Supplementary Information:**

The online version contains supplementary material available at 10.1186/s12934-021-01691-3.

## Background

*Saccharomyces cerevisiae* is a widely used microbial cell factory to produce recombinant proteins, natural and unnatural products, biofuels and biochemicals [1, 2]. Construction of biosynthetic pathways often requires fine regulation of the expression of multiple genes to balance the intricate metabolic pathway and to achieve high yield for desired products. It is well-known that gene expression in *S. cerevisiae* is controlled by multiple different regulatory elements, including promoters, activators and suppressors, as well as other regulators, which have been extensively engineered for pathway optimizations [3, 4].

In *S. cerevisiae*, a set of native promoters has been well-characterized, including constitutive promoters, which sustain stable expression levels across different growth conditions, and inducible promoters which vary their strengths in response to internal or external stimuli. Strong constitutive promoters are frequently used for driving protein overexpression, such as P_TDH3_, P_TEF1_, P_PGK1_, P_TPI1_, P_ENO2_ [5, 6], Commonly used inducible promoters include galactose-inducible promoters, such as P_GAL1_, P_GAL2_, P_GAL7_, and P_GAL10_. These promoters have been broadly applied in metabolic engineering [7, 8], thus their regulation has been extensively studied and engineered.

The genes involved in the GAL network are divided into two categories, one is related to galactose metabolism and includes *GAL1*, *GAL2*, *GAL7* and *GAL10*, and the other is responsible for the regulation of the former including *GAL4*, *GAL80*, *GAL3*. Gal4p is the transcriptional activator, Gal80p is a repressor of Gal4p. In general, Gal4p docks on the GAL promoters. In the absence of galactose, Gal80p binds to Gal4p and inhibits the activation of GAL promoters. When galactose is added, Gal3p is a transcriptional regulator that forms a complex with Gal80p to relieve Gal80p inhibition of Gal4p and activates the activity of the GAL promoters. However, this activation could be hindered by glucose repression [9]. *GAL1*, a gene encoding a galactokinase to covert galactose into galactose-1-phosphate, has also be knocked out to inhibit galactose metabolism so that galactose became a gratuitous inducer for galactose-inducible promoters [10, 11]*.* Gal80p has been deleted to allow all GAL promoters to be functional under diverse carbon sources because it is not economically feasible to use galactose as a sole carbon source for the fermentation of many products [12, 13].

More recently, promoter engineering has been utilized to construct synthetic promoters for various applications. It has been found that synthetic promoters could be created by combining of core promoters, which directly interact with RNA polymerase II (Pol-II) and other general transcription factors, and upstream activating sequences (UASs), which improve promoter activity and increase protein production. The UASs include UAS_CLB_ from the *CLB2* promoter, UAS_CIT_ from the *CIT1* promoter, UAS_ENO_ from the *ENO2* promoter, and UAS_GAL_ from the *GAL1* promoter [14, 15]. However, few studies have been performed to systematically characterize the synergy between UASs and core promoters or the role of other endogenous regulatory elements on the resulting synthetic promoters. In this study, a library of synthetic GAL promoters using different UASs and core promoters was built to investigate their interaction. Furthermore, a synthetic promoter system was developed by engineering endogenous GAL regulators into synthetic promoters, and this promoter system could control protein production with improved dynamic range in *S. cerevisiae* grown in different carbon sources.

## Results and discussion

### Engineering the upstream activating sequences of *GAL1* promoter

**Fig. 1:**
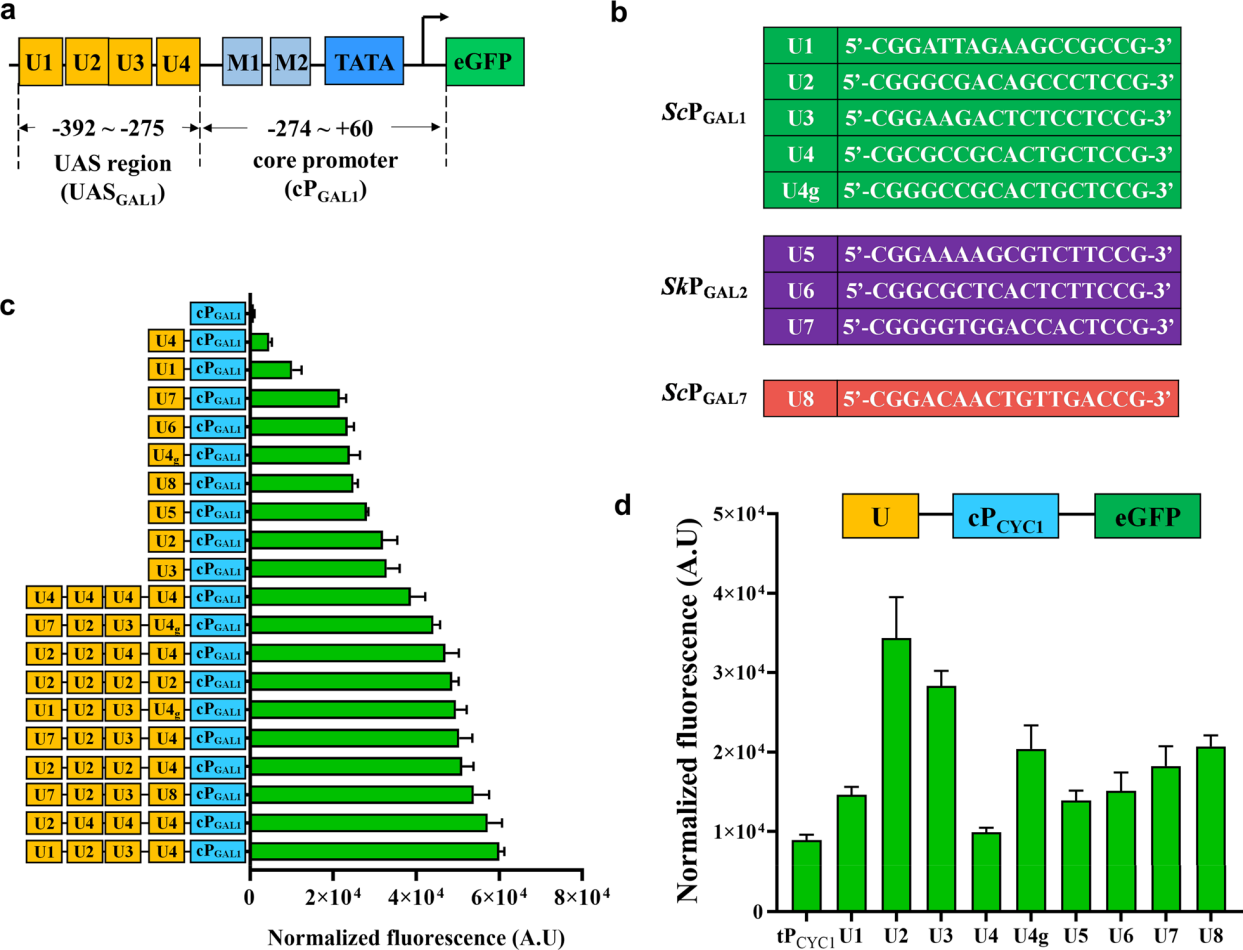
UASs and their impact on *GAL* promoter strength in *S. cerevisiae.* **a** The architecture of the native *GAL1* promoter. Upstream activating sequences (UAS_GAL1_) and core promoter (cP_GAL1_) are shown. U1, U2, U3, and U4 represented the four UASs of P_GAL1_; M1 and M2 represent repressor Mig1p binding sites, which was responsible for glucose repression. **b** The sequence of UASs used in this study. U1-U4 and U4g are from *Sc*P_GAL1_ (*S. cerevisiae*, green), U5-U7 from *Sk*P_GAL2_ (*S. kudriavzevii*, purple) and U8 from *Sc*P_GAL7_ (*S. cerevisiae*, red). (c-d) The effect of UASs on the activities of synthetic promoters when cP_GAL1_ (**c**) and cP_CYC1_ (**d**) were used as core promoter. The activities of all synthetic promoters were tested after induction with 2% galactose for 24 h. Normalized fluorescence = Fluorescence intensity/OD_600_. Data are mean ± SD (standard deviation) from three biological replicates

Herein, we divided the *GAL1* promoter (P_GAL1_) into an UAS region (UAS_GAL1_) and a core promoter (cP_GAL1_) for study, as shown in Fig. 1A. UAS_GAL1_ is the region containing four UASs designated as U1, U2, U3, U4, while cP_GAL1_ was used to represent the sequence between UAS region and the start codon. In a previous study, it was shown that UASs of *GAL1* promoter had different activities [16], however, their individual roles in promoter strength have not yet been elucidated. In this study, to understand how UASs work on *GAL1* promoter, we firstly analyzed several UASs (Fig. 1B), including four UASs from *GAL1* promoter, the most widely used GAL promoter, one from *GAL7* promoter, which contains one single strong UAS (U8), and three (U5, U6 and U7) from *Saccharomyces kudriavzevii GAL2* promoter (*Sk*P_GAL2_), the strongest GAL promoter in the literature [17]. In addition, the non-conserved CGC triplet at the 5′ terminus of U4 was mutated to the conserved CGG triplet and named U4g (Fig. 1B). These UASs were fused directly to the 5′-end of cP_GAL1_ (Fig. 1C) and cP_CYC1_, the core promoter of *CYC1* (Fig. 1D) [14] to obtain a series of synthetic promoters. The gene encoding green fluorescent protein (eGFP) was then placed under the control of these synthetic promoters as a sensor to monitor their activities [18].

As shown in Fig. 1C, core promoter cP_GAL1_ showed low basal activity in synthetic complete (SC) medium containing 2% galactose, while the addition of every single UAS improved the promoter activities under the same condition. Addition of U2 and U3 resulted in the highest activities which were 36.5-fold and 37.6-fold higher than cP_GAL1_, respectively. The fusion of U1 and U4 also had a positive effect on the promoter strength, about 10.9-fold and 4.5-fold higher compared with cP_GAL1_ respectively, although these activities were significantly lower than that of the U2-U3 fusion. The activity of U4g-cP_GAL1_ was 4.6-fold greater than U4-cP_GAL1_, indicating the conserved CGG triplet was important for UAS activity. Moderate activities were observed for the synthetic promoters with U8 and heterologous U5, U6, and U7. When cP_CYC1_ was used as the core promoter, the effect of each UAS showed a similar pattern as in the case of cP_GAL1_ (Fig. 1D). UAS engineering did not alter the basal activities of the synthetic promoters when cells were grown in SC medium with 2% glucose as compared to cP_GAL1_ and cP_CYC1_ (Additional file 1: Fig S1A and S1B). These results illustrated that individual UASs could be used to fine-tune the promoter activity.

To analyze the synergistic effect of UASs, we used P_GAL1_ as a model to create single and multiple UAS-negative constructs with minimal perturbation of the promoter sequences by mutating the conserved 5′ terminal CGG triplet to AGG, which resulted in a sharp reduction in promoter activity for one or more UASs (Additional file 1: Fig S1C). As shown in Additional file 1: Fig S1D, mutation of a single UAS led to 10% to 18% reduction of the promoter strength while the synthetic promoter with mutations in all four UASs, U4m, had 50% of the activity of P_GAL1_. Mutations in U4m was recovered individually to study the individual role of each UAS in the complex. The results showed that recovery of U2 or U3 did not have a significant change in the promoter activity, while U1 and U4 led to a significantly higher activity. These results were not in line with single UAS fusions, indicating that U1 and U4 play essential roles in *GAL1* promoter. We further investigated the UAS region of the *GAL1* promoter by replacing U1 and U4, two weak UASs in the original construct, with U7, U4g, and/or U8, stronger UASs, to afford a few synthetic promoters. However, similar or lower activities were observed for these synthetic promoters. We also replaced all four UASs with U4, the weakest UAS in P_GAL1_. The resulting promoter showed low basal activity. Partially replacement of U4s with U2 generated stronger promoters, while the activity did not correlate to the number of U2s. These results indicated there was no direct linkage between individual UASs and the resulting promoter activity (Fig. 1C). A previous study showed that U1 and U4 may be not involved in promoter activation but take part in a larger regulatory mechanism that regulated equal expression of Gal1p and Gal10p [19]. Thus, substitutions of U1 and U4 would probably disrupt this large regulatory mechanism and result in a decrease of P_GAL1_ activity. By engineering the sequence, number, and substitution of UASs, we created a series of galactose-inducible promoters with different strengths which could be used for future engineering work (Fig. 1C) and these promoters displayed low basal expression under glucose growth condition (Additional file 1: Fig S1A). These results demonstrated that a single UAS was important but not determinant to P_GAL1_ activity, indicating that the *GAL1* UASs may have subtle synergistic effects and be involved in other positive regulation mechanisms which would be destroyed when engineered.

### The function of core promoter on *GAL1* promoter

**Fig. 2:**
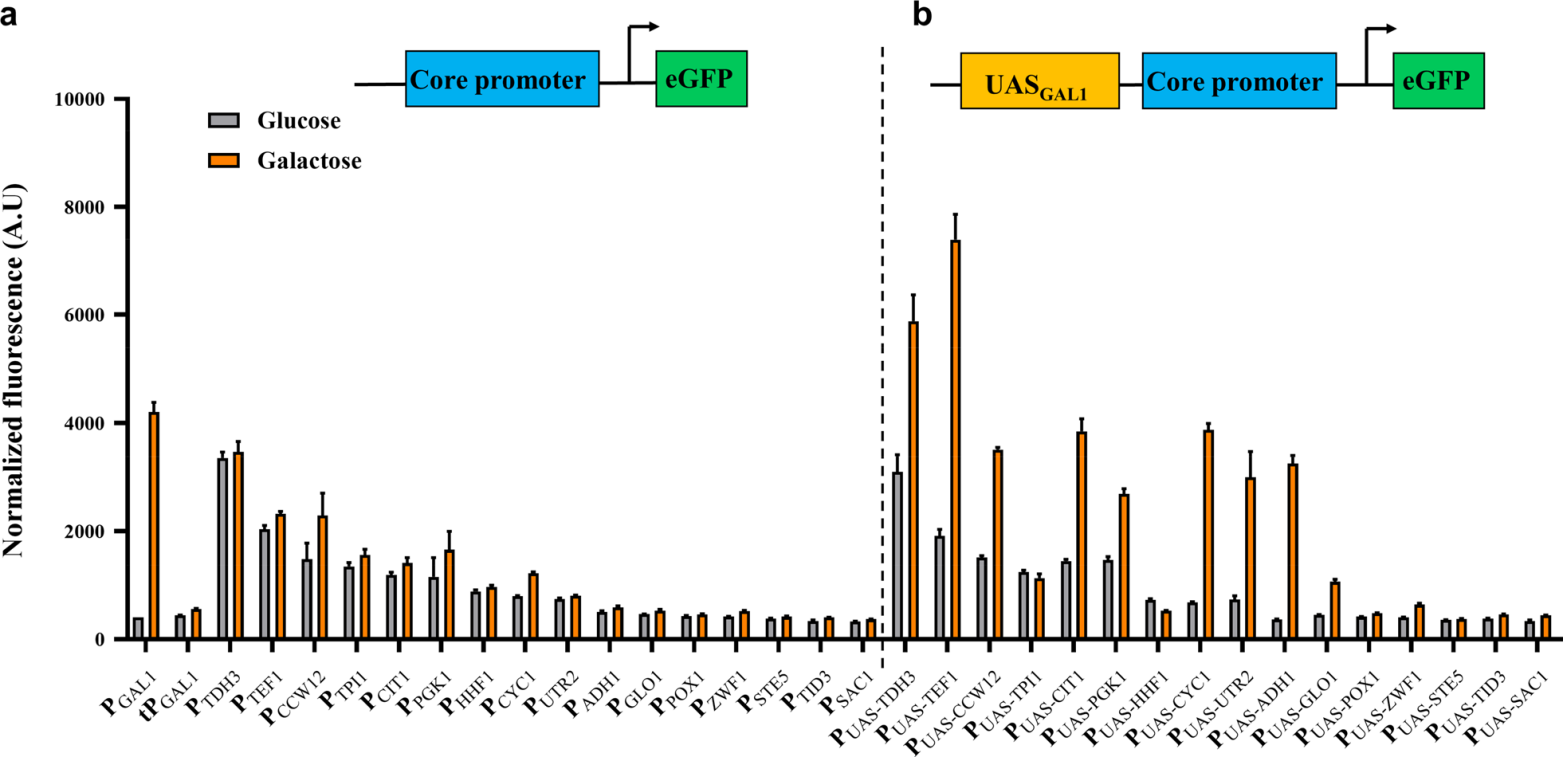
Characterization of the synthetic promoters by core promoter replacement. The normalized fluorescence for constructs with only the core promoter (**a**) and with UAS_GAL1_ fusion (**b**). The activities of all synthetic promoters were tested after cultivation with 2% glucose (grey) or galactose (orange) for 24 h. Data are mean ± SD from three biological replicates

We then tried to replace the core promoter region to further explore the interplay of UASs and core promoters. Several constitutive promoters with a dynamic range of activity were used to replaced cP_GAL1_; their sequences can be found in Additional file 1: Table S1 [5, 18]. The activities of the selected promoters were similar as previously reported in the presence of 2% glucose, and no significant activity difference could be observed when galactose was used as the sole carbon source (Fig. 2A). As shown in Fig. 2B, when cP_GAL1_ was switched with the core regions of several constitutive promoters, more than half of the resulting synthetic promoters became galactose-inducible as expected. The activities of P_UAS-TDH3_ and P_UAS-TEF1_, in which strong constitutive promoter P_TDH3_ and P_TEF1_ was used, were 30% and 68% higher than P_GAL1_ under 2% galactose, respectively, whereas the activity of P_UAS-CYC1_ and P_UAS-CIT1_ were comparable to P_GAL1_ in the same condition. However, some of the core promoter substitutions did not respond to galactose induction, such as P_UAS-TPI1_, P_UAS-HHF1_, P_UAS-POX1_, and P_UAS-STE5_, despite the fact that P_TPI1_ and P_HHF1_ showed stronger activity than cP_GAL1_ under 2% galactose, indicating that the upstream activating sequence region may have some specificity for the core promoter. When glucose was used as the sole carbon source, most of the synthetic promoters maintained the same expression levels as their corresponding core promoters, indicating that combinatorial interaction between UAS_GAL1_ and core promoter did not drastically affect the native properties of core promoters. These results show that galactose-inducible promoters stronger than P_GAL1_ can be created by fusing UASs to the core promoters, which significantly expanded the dynamic range of galactose inducible promoters by more than 50% over previously reported maximum activity [14].

### *GAL80* deletion increased synthetic promoter activity under galactose and glucose growth condition

**Fig. 3:**
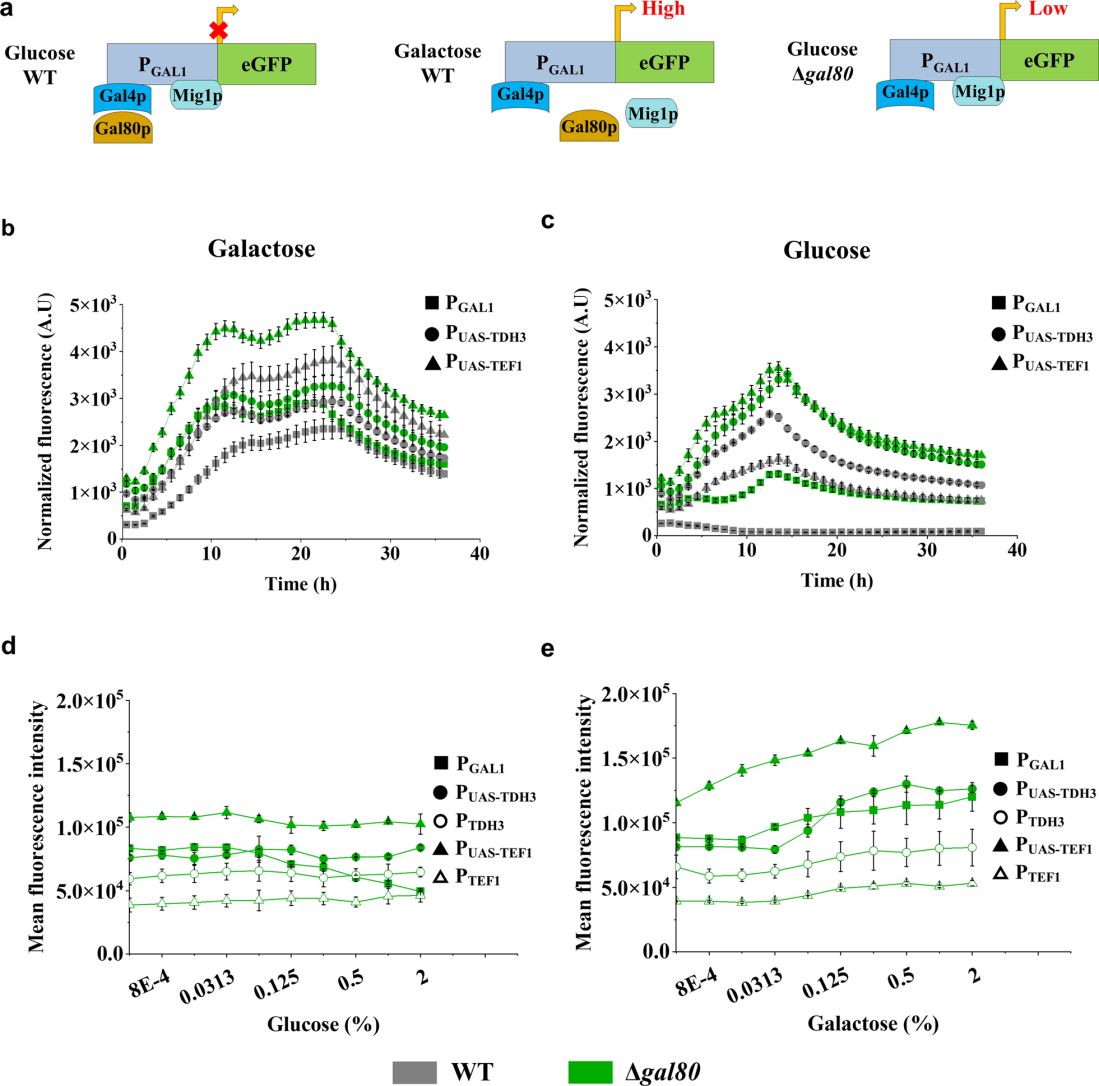
*GAL80* deletion affected the activities of synthetic promoters under different carbon sources. **a** The regulation mechanism of *GAL1* promoter under galactose and glucose condition. Gal4p: the transcriptional activator responsible for galactose induction; Gal80p: the repressor of Gal4p; Mig1p: the repressor that responds to glucose. WT: Wildtype strain; Δ*gal80*: *GAL80* deletion strain. **b**, **c** *GAL80* deletion improved the activities of *GAL* promoters in the presence of 2% galactose (b) or 2% glucose (**c**). The promoter activities were continuously monitored for 36 h. **d** Glucose inhibition profile of promoters. Synthetic promoters were incubated with 2% raffinose with a glucose gradient from 0 to 2% for 5 h with initial OD_600_ at 0.1. After inhibition, mean fluorescence intensity of cells was analyzed by flow cytometry. **e** Galactose induction profile of native and synthetic promoters. 2% raffinose was used as background carbon source with galactose gradient concentration from 0 to 2% for 5 h with initial OD600 at 0.1. After induction, mean fluorescence of intensity of cells was analyzed by flow cytometry. Data are mean ± SD from three biological replicates and the shadow patterns of the curve represents errors as standard deviation

To analyze how the synthetic promoters interact with the endogenous regulator, Gal80p, the constructs with eGFP driven by P_GAL1_, P_UAS-TDH3_, and P_UAS-TEF1_ were evaluated in both the wildtype and the *GAL80* deletion strains in the presence of 2% galactose or glucose (Fig. 3A). Cell density and fluorescence were continuously monitored for 36 h to accurately characterize the host growth rate as well as the promoter strength in the presence of these carbon sources. As shown in Fig. 3B, deletion of *GAL80* further increased the activity of P_GAL1_, P_UAS-TDH3_, and P_UAS-TEF1_ in the presence of 2% galactose. The maximal activity of P_GAL1_ in the *GAL80* deletion strain was improved by 23% compared to that of a wildtype strain, whereas the activities of P_UAS-TEF1_ and P_UAS-TDH3_ increased by 23% and 11%, respectively, while no change in the growth rate was observed (Additional file 1: Fig S2A). Thus, we speculated that, even in the presence of 2% galactose, apo-Gal80p still presented in a concentration that could interact with Gal4p to suppress its activity, whereas deletion of Gal80p released all Gal4p to act as activator, leading to the improvement of the corresponding promoters’ activity.

We then compared the activities of these promoters in strains with or without Gal80p in the presence of 2% glucose. As shown in Fig. 3C, P_GAL1_ showed a dramatic increase of activity in the *GAL80* knockout strain as expected, while the maximal activities of P_UAS-TDH3_ and P_UAS-TEF1_ were increased by 32% and 120% in the *GAL80* deletion strain compared to those in the wildtype strain. In addition, *GAL80* deletion did not affect host growth on glucose (Additional file 1: Fig S2B). It is interesting to notice that all tested promoters in the *GAL80* deletion strains had lower activities in the presence of 2% glucose compared with that in the presence of 2% galactose, especially for P_GAL1_ where a 54% decrease was observed (Fig. 3B, C). The dramatic decrease in P_GAL1_ activity even in *GAL80* knockout strain is presumably because of glucose inhibition through the repressor Mig1p binding sites within the core promoter cP_GAL1_ (Fig. 3A). Mig1p is a transcriptional repressor that responds to glucose and binds to a consensus sequence 5′-SYGGGG-3′ [20]. P_GAL1_ contains two Mig1p binding sites [21]. According to sequence analysis, the synthetic promoters P_UAS-TDH3_ and P_UAS-TEF1_ do not contain Mig1p binding sites and thus should escape glucose repression. By testing these two promoters in glucose concentrations between 0 and 2%, we found that P_GAL1_ activity in the *GAL80* deletion strain decreased when the glucose concentration was higher than 0.0625%, which was not observed in the case of P_UAS-TDH3_ and P_UAS-TEF1_. It confirmed that two synthetic promoters did not suffer from glucose repression (Fig. 3D).

We examined the activity of *GAL* and core promoters in the presence of different galactose concentrations with flow cytometry and measured the mean fluorescence intensity as well as the cell population with active transcription (ON cell). The results revealed that the percentage of ON cells for P_GAL1_ in the wildtype strain increased with increasing galactose concentration which was not observed in the *GAL80* deletion strain (Additional file 1: Fig S3A). The percentage of ON cells in the populations for P_UAS-TDH3_ and P_UAS-TEF1_ were similar in both the wildtype and *GAL80* deletion strain and not affected by different galactose concentration (Additional file 1: Fig S3B), indicating that the higher activity of promoters in the presence of galactose in the *GAL80* deletion strains were not due to the activation of more cells, but rather the increase of mean transcriptional level. Further analysis of the mean fluorescence intensity revealed that the expression levels driven by P_GAL1_, P_UAS-TDH3_ and P_UAS-TEF1_ were positively correlated with the galactose concentrations in both wildtype strain and *GAL80* deletion strains, but showed no such correlation with constitutive promoters P_TDH3_ and P_TEF1_ (Additional file 1: Fig S3C and Fig. 3E), demonstrating that galactose may have an additional role in increasing the activity of galactose-inducible promoters even in the *GAL80* deletion strain.

### Double deletion of *GAL80* and *GAL1* acquired high promoter activity under different carbon sources

**Fig. 4:**
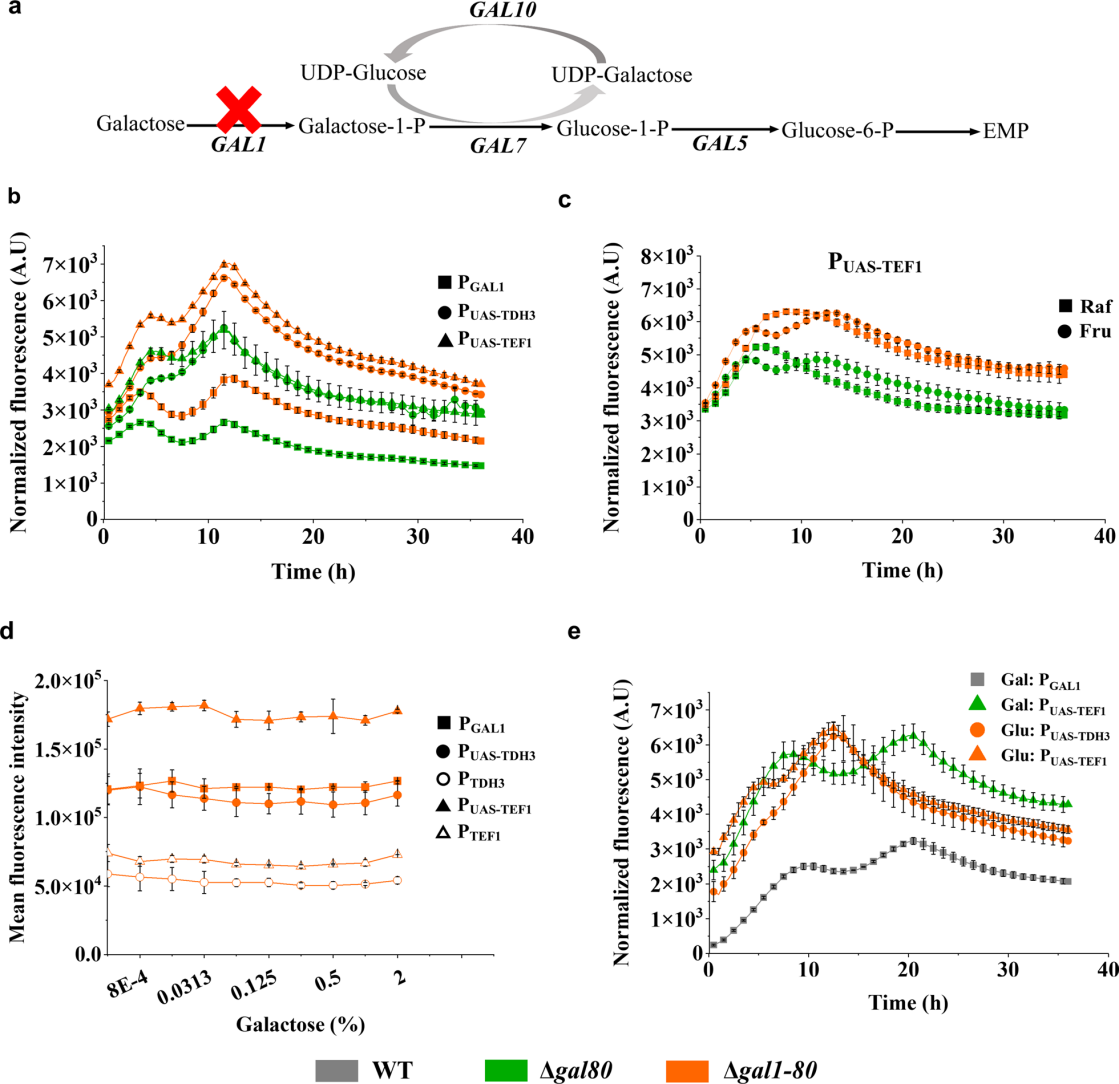
The effect of *GAL1* and *GAL80* double deletion on synthetic promoters. **a** Schematic diagram of galactose metabolism. *GAL1*: Galactokinase; *GAL7*: Galactose-1-phosphate uridyl transferase; *GAL10*: UDP-glucose-4-epimerase; *GAL5*: Phosphoglucomutase. UDP: Uridine diphosphate; EMP: Embden-Meyerhof-Parnas. In this study, *GAL1* was deleted to block galactose metabolism. **b** Double deletion of *GAL1* and *GAL80* enhanced *GAL* promoter activity under glucose growth condition. **c** Double deletion of *GAL1* and *GAL80* improved synthetic promoter activity under raffinose or fructose conditions. **d** Galactose induction profile of promoters in *GAL1*/*GAL80* double deletion strain. **e** Characterization of the synthetic promoter system under different carbon sources. Data are mean ± SD from three biological replicates and the shadow patterns of the curve represents errors as standard deviation

We then deleted *GAL1* in *GAL80* deletion strain to block galactose metabolism so that galactose would be a gratuitous inducer (Fig. 4A). Unanticipatedly, we observed that P_UAS-TDH3_ and P_UAS-TEF1_ in the *GAL1/GAL80* double deletion strain had 35% higher activity than in the *GAL80* single deletion strain under 2% glucose condition (Fig. 4B). The same effects can also be observed with other carbon sources, including fructose and raffinose, whereas the constitutive promoters such as P_TEF1_ did not have any improvement (Fig. 4C and Additional file 1: Fig S4A). However, their activities were no longer affected by galactose in the *GAL1*/*GAL80* double deletion strains (Additional file 1: Fig S4B). The galactose inducible profile showed that the percentage of ON cells and the expression levels driven by all promoters were not affected by additional galactose in the double deletion strains (Additional file 1: Fig S3D and 4D). These results indicate that double deletion of *GAL80* and *GAL1* could completely relieve the galactose dependence of galactose-inducible promoters, thereby allowing them to have maximal activities on multiple carbon sources. In all, compared to P_GAL1_ in the wildtype strain, the maximal activity of P_UAS-TDH3_ and P_UAS-TEF1_ in the double deletion strains increased by about 100% under both galactose and glucose growth conditions, with an expanded dynamic range of promoter activity under other carbon sources as well (Fig. 4E).

### The efficient promoter system enhanced β-glucosidase expression

**Fig. 5:**
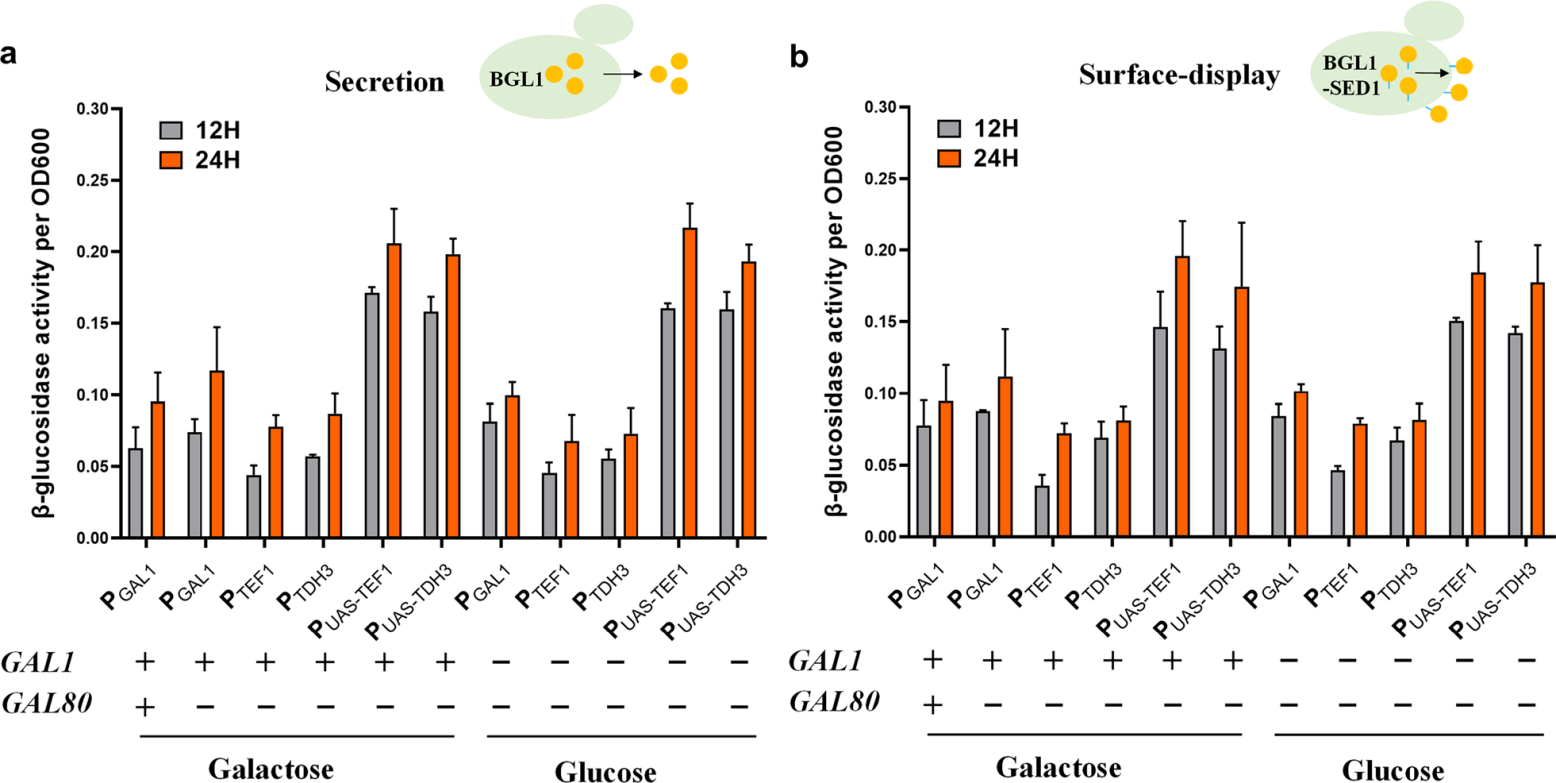
The synthetic promoter system significantly improved β-glucosidase secretion (**a**) and surface display (**b**). *SED1*, a gene encoding yeast cell wall protein which is a commonly used surface-displayed system. Enzyme activities were measured under glucose or galactose growth conditions for 12 h and 24 h, respectively. + represents in the presence of *GAL1* or *GAL80*;—represents the deletion of *GAL1* or *GAL80*.The enzyme activity was quantified by *p*NPG assay and the data are mean ± SD from three biological replicates

To test the utility of the synthetic promoters, secretory and surface-displayed expression of β-glucosidase (BGL1) and a cellulase responsible for degrading cellulose into glucose were used as examples. As shown in Fig. 5A, compared to the secretion of BGL1 driven by P_GAL1_ in wildtype strain, BGL1 under control of P_UAS-TEF1_ in the *GAL80* deletion strain was more than two-folder higher in the presence of galactose, and its secretion driven by P_UAS-TDH3_ or P_UAS-TEF1_ in the *GAL80* and *GAL1* double deletion strains was more than two-folder higher under glucose growth condition. The surface-display of BGL1 exhibited nearly identical expression pattern as in secretion (Fig. 5B). These results illustrate that the synthetic promoters could increase intracellular expression and extracellular secretion of recombinant proteins both under glucose and galactose growth condition.

## Conclusions

In summary, our results demonstrate that engineering P_GAL1_ enabled creation of synthetic galactose-inducible promoters with an expanded dynamic range, and it is the first reported synthetic promoter in *S. cerevisiae* with a twofold higher activity than P_GAL1_ under a variety of different carbon sources. The function of each element of these promoters was analyzed; we found that the upstream activating sequences UAS_GAL1_ are important to the inducibility of synthetic promoters and subtle synergistic effects within the UAS region are destroyed when the UASs are perturbed. When considering the core promoter region, stronger core promoters tend to produce stronger synthetic promoters. Furthermore, a system was developed for well-controlled protein expression in *S. cerevisiae* under different carbon sources. We found that deletion of *GAL80* could further strengthen galactose-inducible promoter activities under galactose growth conditions, and double deletion of *GAL80* and *GAL1* could completely relieve the galactose dependence of these synthetic promoters derived from *GAL1* to thereby unleash their maximal activities on different carbon sources.

## Materials and methods

### Strains and media

*Escherichia coli* Trans5a was used for plasmid construction and propagation, and its culture medium was LB (10 g/L tryptone, 5 g/L Yeast extract, 10 g/L NaCl) with or without ampicillin. *S. cerevisiae* CEN. PK2-1C was used as the host for testing promoters’ activities and expressing recombinant proteins. CEN. PK2-1C was grown in YPD (20 g/L peptone, 10 g/L yeast extract and 20 g/L glucose). The eGFP expressing strains were cultivated in SC medium, and the BGL1 expressing strains were cultivated in SC-SCAA medium [21], 2% of glucose or galactose was added according to the experimental requirements.

### Plasmid construction

The primers used in this study were shown in Additional file 1: Table S2. The yeast centromeric plasmid pPOT2 containing the *URA3* gene as a marker was used as the backbone. The *GAL1* promoter amplified from the commercial plasmid pYD1 (Invitrogen), the e*GFP* gene and the *ADH1* terminator were inserted into pPOT2 by Gibson assembly and the recombinant plasmid named P_GAL1_-eGFP. The promoter cP_GAL1_ and cP_CYC1_ fused with or without UASs were cloned to P_GAL1_-eGFP though P_GAL1_ replacement. Point mutation of UASs were constructed from P_GAL1_-eGFP by QuickChange mutagenesis. Replacement of UAS sites was achieved by Gibson assembly of mutant fragment and corresponding backbone amplified from P_GAL1_-eGFP. All constitutive promoters were amplified from CEN.PK2-1C genomic DNA and then were used to replace the cP_GAL1_ of P_GAL1_-eGFP, respectively. The coding gene of BGL1 was amplified from the previously study’s plasmid [22]. The recombinant plasmids for expression of BGL1 controlled by P_GAL1_, P_UAS-TEF1_ and P_UAS-TDH3_ were constructed by eGFP replacement.

### *GAL1* and *GAL80* deletion

In order to knock out the *GAL1* and *GAL80*, the pCUT plasmid containing Cas9 gene was used in this study as previously described [5]. The guide RNA and homologous fragments were designed by Yeastriction (http://yeastriction.tnw.tudelft.nl/#!/) and SGD (https://www.yeastgenome.org/), respectively (Additional file 1: Table S3). Linearized pCUT plasmid, guide RNA and homologous fragment were transformed into CEN.PK2-1C for *GAL1* and *GAL80* deletion, respectively.

### Fluorescence measurement

Three clones of each strain were placed in 300 μL SC-URA (2% glucose) medium for 24 h (96-well plate, 800 rpm), and then transferred into SC-URA (2% glucose or galactose) with initial OD_600_ at 0.2. After 24 h of culture, the GFP fluorescence were measured by microplate readers (Tecan, Infinite® 200 PRO), the excitation at 488 nm and the emission at 520 nm. Continuous monitoring of fluorescence and growth was cultured in an enzyme-labeled instrument for 36 h.

### Galactose or glucose response measurements

In this experiment, 2% raffinose was used as a background carbon source. Colonies were cultured in SC medium (2% raffinose). After the overnight cultivation, cells were inoculated into in 2% raffinose and galactose (or glucose) with concentration from 0 to 2% to induce for 5 h with initial OD_600_ at 0.1. After induction, cells were collected and resuspended with PBS, and then the fluorescence distribution and the mean fluorescence intensity of 30,000 cells in each sample was recorded by flow cytometry (CytoFLEX S, Beckman Coulter). According to the fluorescence distribution, cells were divided into two populations of active transcription (ON cell) and inactive transcription (OFF cell) [23].

### Enzyme assays

The strains expressing BGL1 were inoculated into SC-SCAA (2% glucose or galactose) medium and grown for 24 h. BGL1 activity was detected using *p*-nitrophenyl-β-D-glucopyranoside *p*NPG as the substrate, as described previously [22]. Enzymes were incubated in 50 mM citrate buffer (pH 5.0) with 5 mM *p*NPG at 50 °C for 30 min. Sodium carbonate (10%, w/v) was added to stop the reaction, and the absorbance was measured at 405 nm. One unit of the BGL1 activity was defined as the amount of enzyme that released 1 μmol of *p*NP from the substrate per minute at 50 °C.

## Supplementary Information

**Additional file 1: Fig. S1.** Engineering UASs affected the activities of GAL promoters. (a-b) The effect of UAS on the activities of synthetic promoters by using cPGAL1 (a) or cPCYC1 (b) as core promoter under glucose growth condition. (c) The importance of the conserved 5’ terminal CGG triplets on UAS. (d) UASs regulated the GAL1 promoter activity. **Fig. S2.** The growth curves under galactose (a) and glucose (b) cultivation conditions. **Fig. S3.** Galactose induction profile of promoters. (a-b) The fraction of ON cells driven by different promoters in WT (a) and △gal80 (b). 2% raffinose was used as background carbon source with galactose gradient concentration from 0% to 2% for induction. For bimodal expression profiles, cells were divided into two populations of active transcription (ON cell) and inactive transcription (OFF cell), the fraction of cells occupying the ON-state is defined as promoter inducibility and the mean fluorescence intensity of ON cells represents promoter strength. (c) The mean fluorescence intensity of ON cell in WT. (d) The fraction of ON cells in Δgal1-80. The solid pattern indicates the promoter with UASGAL1, and the hollow pattern indicates the promoter without UASGAL1. **Fig. S4.** The effect of GAL80 and GAL1 double deletion on PTEF1 activity in various carbon sources. (b) The effect of additional galactose concentration on the activity of synthetic GAL promoter PUAS-TEF1 in Δgal80 and Δgal1-80. 2% glucose was used as carbon source with galactose gradient concentration from 0% to 2% for induction. **Table S1.** Sequences of constitutive promoters used in this study. **Table S2.** Primers used in this study. **Table S3.** Guide RNA and homologous fragments used in this study.

## Data Availability

All data generated or analyzed during this study are included in this published article and its Additional file 1.

## References

[1] Nevoigt E (2008). Progress in metabolic engineering of *Saccharomyces cerevisiae*. Microbiol Mol Biol Rev.

[2] Turanlı-Yıldız B, Hacısalihoğlu B, Çakar ZP (2017). Advances in metabolic engineering of *Saccharomyces cerevisiae* for the production of industrially and clinically important chemicals. Old Yeasts New Quest.

[3] Xu N, Wei L, Liu J (2019). Recent advances in the applications of promoter engineering for the optimization of metabolite biosynthesis. World J Microb Biotechol.

[4] Jin LQ, Jin WR, Ma ZC, Shen Q, Cai X, Liu ZQ, Zheng YG (2019). Promoter engineering strategies for the overproduction of valuable metabolites in microbes. Appl Microbiol Biot.

[5] Reider Apel A, d'Espaux L, Wehrs M, Sachs D, Li RA, Tong GJ, Garber M, Nnadi O, Zhuang W, Hillson NJ, Keasling JD, Mukhopadhyay A (2017). A Cas9-based toolkit to program gene expression in *Saccharomyces cerevisiae*. Nucleic Acids Res.

[6] Partow S, Siewers V, Bjørn S, Nielsen J, Maury J (2010). Characterization of different promoters for designing a new expression vector in *Saccharomyces cerevisiae*. Yeast.

[7] Ro DK, Paradise EM, Ouellet M, Fisher KJ, Newman KL, Ndungu JM, Ho KA, Eachus RA, Ham TS, Kirby J, Chang MC, Withers ST, Shiba Y, Sarpong R, Keasling JD (2006). Production of the antimalarial drug precursor artemisinic acid in engineered yeast. Nature.

[8] Luo X, Reiter MA, d'Espaux L, Wong J, Denby CM, Lechner A, Zhang Y, Grzybowski AT, Harth S, Lin W, Lee H, Yu C, Shin J, Deng K, Benites VT, Wang G, Baidoo EEK, Chen Y, Dev I, Petzold CJ, Keasling JD (2019). Complete biosynthesis of cannabinoids and their unnatural analogues in yeast. Nature.

[9] Johnston M, Flick JS, Pexton T (1994). Multiple mechanisms provide rapid and stringent glucose repression of GAL gene expression in *Saccharomyces cerevisiae*. Mol Cell Biol.

[10] Štagoj MN, Comino A, Komel R (2006). A novel GAL recombinant yeast strain for enhanced protein production. Biomol Eng.

[11] Hovland P, Flick J, Johnston M, Sclafani RA (1989). Galactose as a gratuitous inducer of GAL gene expression in yeasts growing on glucose. Gene.

[12] Wang F, Lv X, Xie W, Zhou P, Zhu Y, Yao Z, Yang C, Yang X, Ye L, Yu H (2017). Combining Gal4p-mediated expression enhancement and directed evolution of isoprene synthase to improve isoprene production in *Saccharomyces**cerevisiae*. Metab Eng.

[13] Westfall PJ, Pitera DJ, Lenihan JR, Eng D, Woolard FX, Regentin R, Horning T, Tsuruta H, Melis DJ, Owens A, Fickes S, Diola D, Benjamin KR, Keasling JD, Leavell MD, McPhee DJ, Renninger NS, Newman JD, Paddon CJ (2012). Production of amorphadiene in yeast, and its conversion to dihydroartemisinic acid, precursor to the antimalarial agent artemisinin. Proc Natl Acad Sci USA.

[14] Blazeck J, Garg R, Reed B, Alper HS (2012). Controlling promoter strength and regulation in *Saccharomyces cerevisiae* using synthetic hybrid promoters. Biotechnol Bioeng.

[15] Wang J, Zhai H, Rexida R, Shen Y, Hou J, Bao X (2018). Developing synthetic hybrid promoters to increase constitutive or diauxic shift-induced expression in *Saccharomyces cerevisiae*. FEMS Yeast Res.

[16] Giniger E, Varnum SM, Ptashne M (1985). Specific DNA binding of GAL4, a positive regulatory protein of yeast. Cell.

[17] Peng B, Wood RJ, Nielsen LK, Vickers CE (2018). An expanded heterologous GAL promoter collection for diauxie-inducible expression in *Saccharomyces cerevisiae*. ACS Synth Biol.

[18] Keren L, Zackay O, Lotan-Pompan M, Barenholz U, Dekel E, Sasson V, Aidelberg G, Bren A, Zeevi D, Weinberger A, Alon U, Milo R, Segal E (2013). Promoters maintain their relative activity levels under different growth conditions. Mol Syst Biol.

[19] Elison GL, Xue Y, Song R, Acar M (2018). Insights into bidirectional gene expression control using the canonical GAL1/GAL10 promoter. Cell Rep.

[20] Gancedo JM (1998). Yeast carbon catabolite repression. Microbiol Mol Biol Rev.

[21] Wittrup KD, Benig V (1994). Optimization of amino acid supplements for heterologous protein secretion in *Saccharomyces cerevisiae*. Biotechnol Tech.

[22] Tang H, Bao X, Shen Y, Song M, Wang S, Wang C, Hou J (2015). Engineering protein folding and translocation improves heterologous protein secretion in *Saccharomyces cerevisiae*. Biotechnol Bioeng.

[23] Hsu C, Scherrer S, Buetti-Dinh A, Ratna P, Pizzolato J, Jaquet V, Becskei A (2012). Stochastic signalling rewires the interaction map of a multiple feedback network during yeast evolution. Nat Commun.

