# Supplementary material for: A synthetic promoter system for well-controlled protein expression with different carbon sources in Saccharomyces cerevisiae
Source: Microb Cell Fact. 2021 Oct 18;20:202. doi: 10.1186/s12934-021-01691-3 (PMC8522093; doi:10.1186/s12934-021-01691-3)
Supplement: Supplementary file 1 — Additional file 1: Fig. S1. Engineering UASs affected the activities of GAL promoters. (a-b) The effect of UAS on the activities of synthetic promoters by using cPGAL1 (a) or cPCYC1 (b) as core promoter under glucose growth condition. (c) The importance of the conserved 5’ terminal CGG triplets on UAS. (d) UASs regulated the GAL1 promoter activity. Fig. S2. The growth curves under galactose (a) and glucose (b) cultivation conditions. Fig. S3. Galactose induction profile of promoters. (a-b) The fraction of ON cells driven by different promoters in WT (a) and △gal80 (b). 2% raffinose was used as background carbon source with galactose gradient concentration from 0% to 2% for induction. For bimodal expression profiles, cells were divided into two populations of active transcription (ON cell) and inactive transcription (OFF cell), the fraction of cells occupying the ON-state is defined as promoter inducibility and the mean fluorescence intensity of ON cells represents promoter strength. (c) The mean fluorescence intensity of ON cell in WT. (d) The fraction of ON cells in Δgal1-80. The solid pattern indicates the promoter with UASGAL1, and the hollow pattern indicates the promoter without UASGAL1. Fig. S4. The effect of GAL80 and GAL1 double deletion on PTEF1 activity in various carbon sources. (b) The effect of additional galactose concentration on the activity of synthetic GAL promoter PUAS-TEF1 in Δgal80 and Δgal1-80. 2% glucose was used as carbon source with galactose gradient concentration from 0% to 2% for induction. Table S1. Sequences of constitutive promoters used in this study. Table S2. Primers used in this study. Table S3. Guide RNA and homologous fragments used in this study. [file 12934_2021_1691_MOESM1_ESM.docx]

***Supplementary Materials***


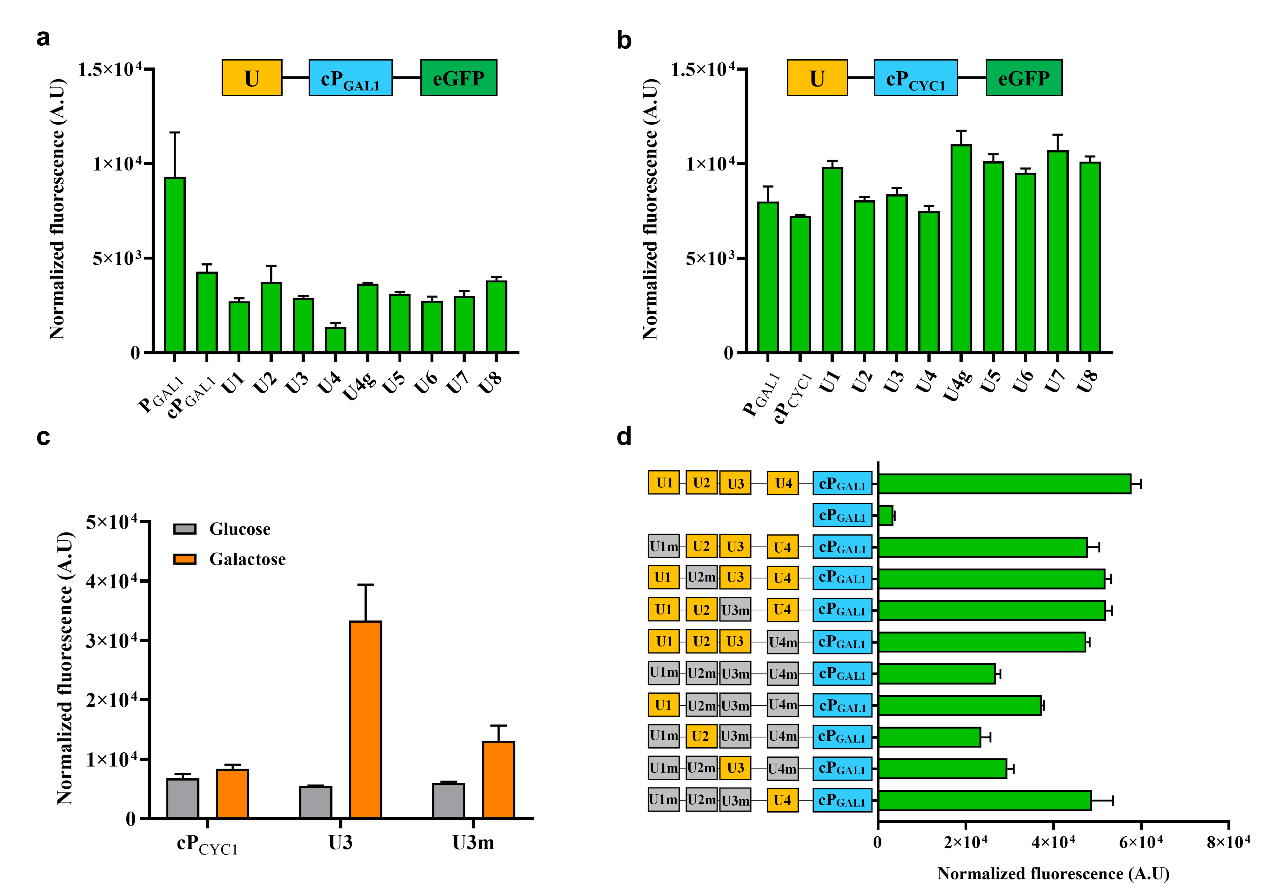


**Supplementary Figure 1** Engineering UASs affected the activities of *GAL* promoters. (a-b) The effect of UAS on the activities of synthetic promoters by using cP_GAL1_ (a) or cP_CYC1_ (b) as core promoter under glucose growth condition. (c) The importance of the conserved 5’ terminal CGG triplets on UAS. U3m: the 5’ terminal CGG of UAS3 was mutated to AGG. (d) UASs regulated the *GAL1* promoter activity. U1m, U2m, U3m, U4m: the 5’ terminal CGG of UAS1, UAS2, UAS3 and UAS4 was mutated to AGG, respectively. Data are mean ± SD (standard deviation) from three biological replicates.


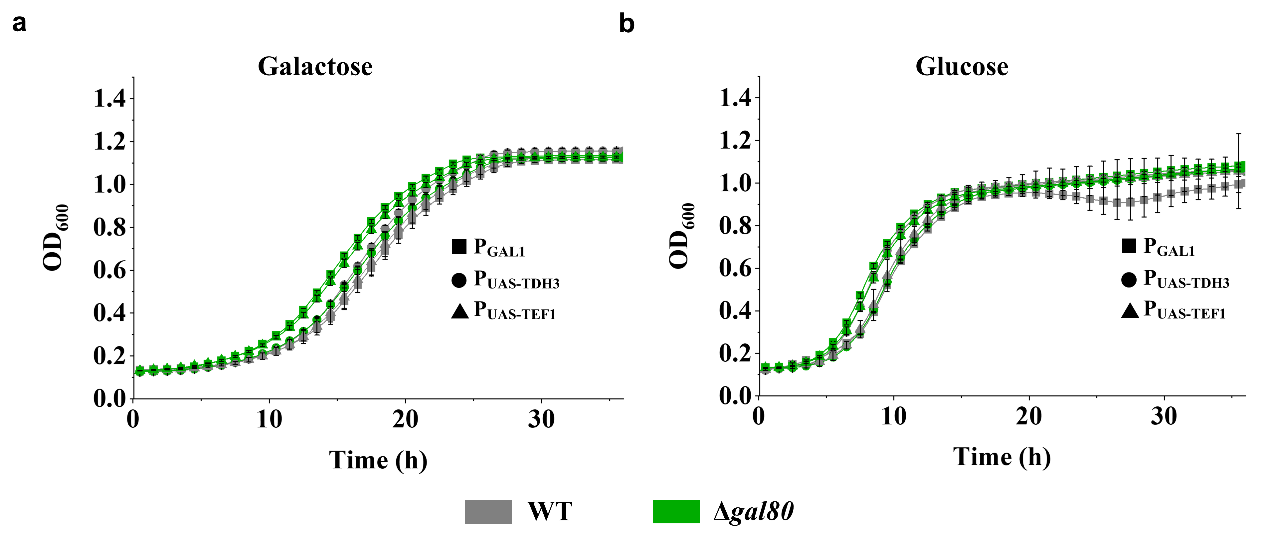


**Supplementary Figure 2** The growth curves under galactose (a) and glucose (b) cultivation conditions.


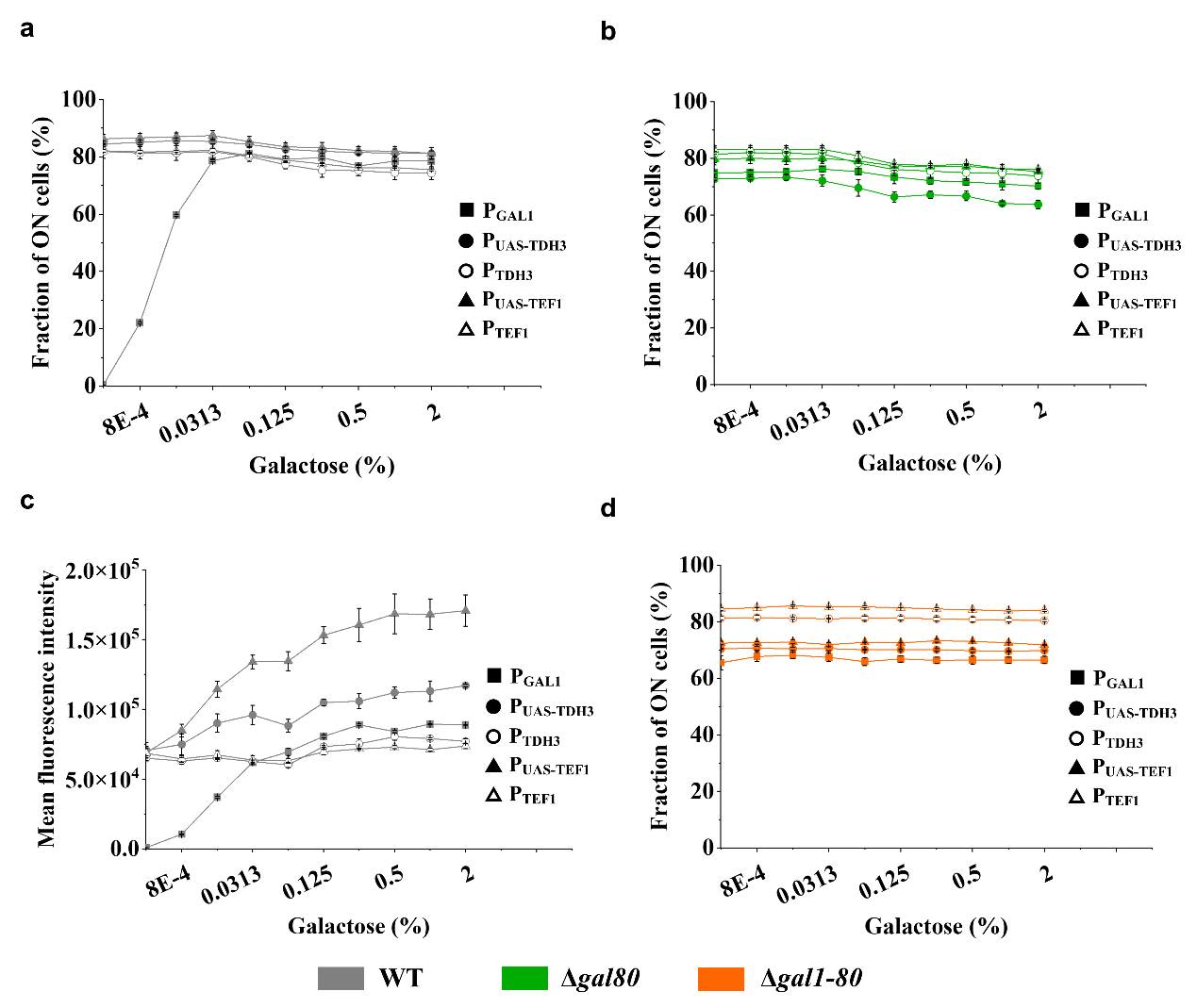


**Supplementary Figure 3** Galactose induction profile of promoters. (a-b) The fraction of ON cells driven by different promoters in WT (a) and *△gal80* (b). 2% raffinose was used as background carbon source with galactose gradient concentration from 0% to 2% for induction. For bimodal expression profiles, cells were divided into two populations of active transcription (ON cell) and inactive transcription (OFF cell), the fraction of cells occupying the ON-state is defined as promoter inducibility and the mean fluorescence intensity of ON cells represents promoter strength. (c) The mean fluorescence intensity of ON cell in WT. (d) The fraction of ON cells in Δ*gal1-80*. The solid pattern indicates the promoter with UAS_GAL1_, and the hollow pattern indicates the promoter without UAS_GAL1_. WT: Wildtype strain; Δ*gal80*: *GAL80* deletion strain. Δ*gal1-80*: *GAL80* and *GAL1* double deletion strain. Data are mean ± SD from three biological replicates.


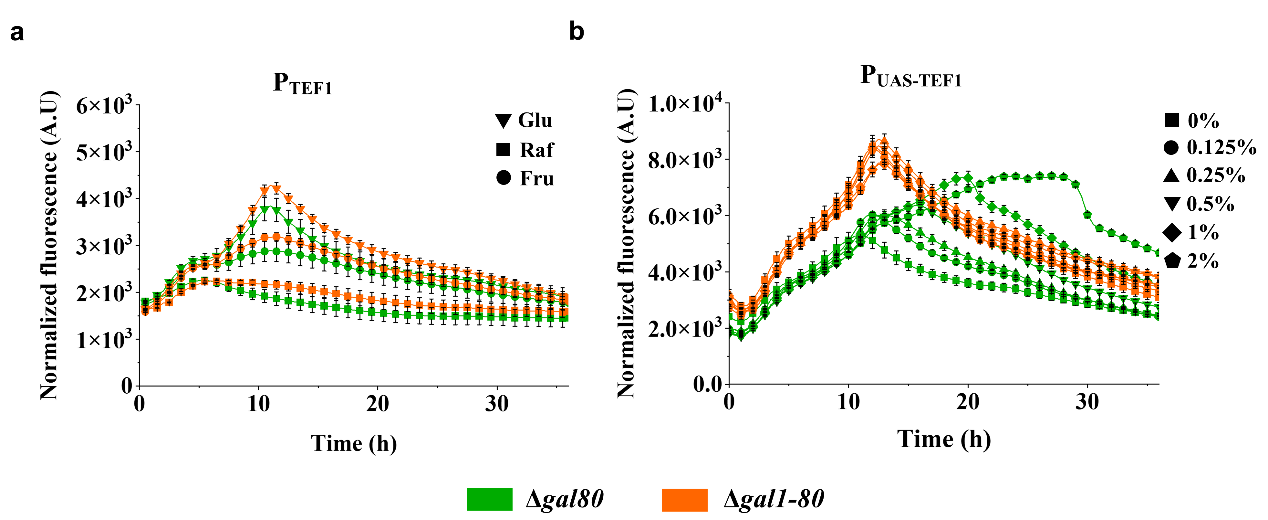


**Supplementary Figure 4** (a) The effect of *GAL80* and *GAL1* double deletion on P_TEF1_ activity in various carbon sources. (b) The effect of additional galactose concentration on the activity of synthetic *GAL* promoter P_UAS-TEF1_ in Δ*gal80* and Δ*gal1-80*. 2% glucose was used as carbon source with galactose gradient concentration from 0% to 2% for induction. Data are mean ± SD from three biological replicates.

Table S1 Sequences of constitutive promoters used in this study.

| Promoter | Sequences (5’-3’) |
| --- | --- |
| UAS_GAL1_ | CGGATTAGAAGCCGCCGAGCGGGcGACAGCCCTCCGAcGGAAGACTCTCCTCCGTGCGTCCTCGTCTTCACCGGTCGCGTTCCTGAAACGCAGATGTGCCTCGCGCCGCACTGCTCCG |
| P_CYC1_ | GAGCTCATTTGGCGAGCGTTGGTTGGTGGATCAAGCCCACGCGTAGGCAATCCTCGAGCAGATCCGCCAGGCGTGTATATATAGCGTGGATGGCCAGGCAACTTTAGTGCTGACACATACAGGCATATATATATGTGTGCGACGACACATGATCATATGGCATGCATGTGCTCTGTATGTATATAAAACTCTTGTTTTCTTCTTTTCTCTAAATATTCTTTCCTTATACATTAGGACCTTTGCAGCATAAATTACTATACTTCTATAGACACGCAAACACAAATACACACACTAA |
| cP_CYC1_ | CTCGAGCAGATCCGCCAGGCGTGTATATATAGCGTGGATGGCCAGGCAACTTTAGTGCTGACACATACAGGCATATATATATGTGTGCGACGACACATGATCATATGGCATGCATGTGCTCTGTATGTATATAAAACTCTTGTTTTCTTCTTTTCTCTAAATATTCTTTCCTTATACATTAGGACCTTTGCAGCATAAATTACTATACTTCTATAGACACGCAAACACAAATACACACACTAA |
| cP_GAL1_ | AACAATAAAGATTCTACAATACTAGCTTTTATGGTTATGAAGAGGAAAAATTGGCAGTAACCTGGCCCCACAAACCTTCAAATTAACGAATCAAATTAACAACCATAGGATGATAATGCGATTAGTTTTTTAGCCTTATTTCTGGGGTAATTAATCAGCGAAGCGATGATTTTTGATCTATTAACAGATATATAAATGGAAAAGCTGCATAACCACTTTAACTAATACTTTCAACATTTTCAGTTTGTATTACTTCTTATTCAAATGTCATAAAAGTATCAACAAAAAATTGTTAATATACCTCTATACTTTAACGTCAAGGAG |
| P_GAL1_ | CGGATTAGAAGCCGCCGAGCGGGCGACAGCCCTCCGACGGAAGACTCTCCTCCGTGCGTCCTCGTCTTCACCGGTCGCGTTCCTGAAACGCAGATGTGCCTCGCGCCGCACTGCTCCGAACAATAAAGATTCTACAATACTAGCTTTTATGGTTATGAAGAGGAAAAATTGGCAGTAACCTGGCCCCACAAACCTTCAAATTAACGAATCAAATTAACAACCATAGGATGATAATGCGATTAGTTTTTTAGCCTTATTTCTGGGGTAATTAATCAGCGAAGCGATGATTTTTGATCTATTAACAGATATATAAATGGAAAAGCTGCATAACCACTTTAACTAATACTTTCAACATTTTCAGTTTGTATTACTTCTTATTCAAATGTCATAAAAGTATCAACAAAAAATTGTTAATATACCTCTATACTTTAACGTCAAGGAG |
| P_TDH3_ | TCATTATCAATACTGCCATTTCAAAGAATACGTAAATAATTAATAGTAGTGATTTTCCTAACTTTATTTAGTCAAAAAATTAGCCTTTTAATTCTGCTGTAACCCGTACATGCCCAAAATAGGGGGCGGGTTACACAGAATATATAACATCGTAGGTGTCTGGGTGAACAGTTTATTCCTGGCATCCACTAAATATAATGGAGCCCGCTTTTTAAGCTGGCATCCAGAAAAAAAAAGAATCCCAGCACCAAAATATTGTTTTCTTCACCAACCATCAGTTCATAGGTCCATTCTCTTAGCGCAACTACAGAGAACAGGGGCACAAACAGGCAAAAAACGGGCACAACCTCAATGGAGTGATGCAACCTGCCTGGAGTAAATGATGACACAAGGCAATTGACCCACGCATGTATCTATCTCATTTTCTTACACCTTCTATTACCTTCTGCTCTCTCTGATTTGGAAAAAGCTGAAAAAAAAGGTTGAAACCAGTTCCCTGAAATTATTCCCCTACTTGACTAATAAGTATATAAAGACGGTAGGTATTGATTGTAATTCTGTAAATCTATTTCTTAAACTTCTTAAATTCTACTTTTATAGTTAGTCTTTTTTTTAGTTTTAAAACACCAAGAACTTAGTTTCGAATAAACACACATAAACAAACAAA |
| P_TEF1_ | GCCGTACCACTTCAAAACACCCAAGCACAGCATACTAAATTTCCCCTCTTTCTTCCTCTAGGGTGTCGTTAATTACCCGTACTAAAGGTTTGGAAAAGAAAAAAGAGACCGCCTCGTTTCTTTTTCTTCGTCGAAAAAGGCAATAAAAATTTTTATCACGTTTCTTTTTCTTGAAAATTTTTTTTTTTGATTTTTTTCTCTTTCGATGACCTCCCATTGATATTTAAGTTAATAAACGGTCTTCAATTTCTCAAGTTTCAGTTTCATTTTTCTTGTTCTATTACAACTTTTTTTACTTCTTGCTCATTAGAAAGAAAGCATAGCAATCTAATCTAAGTTTTAATTACAAA |
| P_CIT1_ | CTAAGAAAAAAGGAGCCATCAAAAACCATTCAGCATTAACTAAAAACGCGGGTAGAGATTACTACATATTCCAACAAGACCTTCGCAGGAAAGTATACCTAAACTAATTAAAGAAATCTCCGAAGTTCGCATTTCATTGAACGGCTCAATTAATCTTTGTAAATATGAGCGTTTTTACGTTCACATTGCCTTTTTTTTTATGTATTTACCTTGCATTTTTGTGCTAAAAGGCGTCACGTTTTTTTCCGCCGCAGCCGCCCGGAAATGAAAAGTATGACCCCCGCTAGACCAAAAATACTTTTGTGTTATTGGAGGATCGCAATCCCTTTGGAGCTTTTCCGATACTATCGACTTATCCGACCTCTTGTTGTTTGAAAATGTCAATTGATATCCATCCATTATATAAATGCTCAAAACTTGCAGCAACTATTCTTTACCCTTCCCCTGTTATGGATTGCTAGTCTTAAGGGGGAAATTTGCTGTTTACTAAAATACAAACCAGGTTTGTTTTGGCTTTTATTTGCATTTAAGTAATTACAATTACAACCATTAAAAAGAAAATAAGGCAAAACATATAGCAATATAATACTATTTACGAAG |
| P_TPI1_ | GAGACCTAACTACATAGTGTTTAAAGATTACGGATATTTAACTTACTTAGAATAATGCCATTTTTTTGAGTTATAATAATCCTACGTTAGTGTGAGCGGGATTTAAACTGTGAGGACCTTAATACATTCAGACACTTCTGCGGTATCACCCTACTTATTCCCTTCGAGATTATATCTAGGAACCCATCAGGTTGGTGGAAGATTACCCGTTCTAAGACTTTTCAGCTTCCTCTATTGATGTTACACCTGGACACCCCTTTTCTGGCATCCAGTTTTTAATCTTCAGTGGCATGTGAGATTCTCCGAAATTAATTAAAGCAATCACACAATTCTCTCGGATACCACCTCGGTTGAAACTGACAGGTGGTTTGTTACGCATGCTAATGCAAAGGAGCCTATATACCTTTGGCTCGGCTGCTGTAACAGGGAATATAAAGGGCAGCATAATTTAGGAGTTTAGTGAACTTGCAACATTTACTATTTTCCCTTCTTACGTAAATATTTTTCTTTTTAATTCTAAATCAATCTTTTTCAATTTTTTGTTTGTATTCTTTTCTTGCTTAAATCTATAACTACAAAAAACACATACATAAACTAAAA |
| P_PGK1_ | GGGCCAGAAAAAGGAAGTGTTTCCCTCCTTCTTGAATTGATGTTACCCTCATAAAGCACGTGGCCTCTTATCGAGAAAGAAATTACCGTCGCTCGTGATTTGTTTGCAAAAAGAACAAAACTGAAAAAACCCAGACACGCTCGACTTCCTGTCTTCCTATTGATTGCAGCTTCCAATTTCGTCACACAACAAGGTCCTAGCGACGGCTCACAGGTTTTGTAACAAGCAATCGAAGGTTCTGGAATGGCGGGAAAGGGTTTAGTACCACATGCTATGATGCCCACTGTGATCTCCAGAGCAAAGTTCGTTCGATCGTACTGTTACTCTCTCTCTTTCAAACAGAATTGTCCGAATCGTGTGACAACAACAGCCTGTTCTCACACACTCTTTTCTTCTAACCAAGGGGGTGGTTTAGTTTAGTAGAACCTCGTGAAACTTACATTTACATATATATAAACTTGCATAAATTGGTCAATGCAAGAAATACATATTTGGTCTTTTCTAATTCGTAGTTTTTCAAGTTCTTAGATGCTTTCTTTTTCTCTTTTTTACAGATCATCAAGGAAGTAATTATCTACTTTTTACAACAAATATAAAACA |
| P_CCW12_ | GGATACTTCATGCTATTTATAGACGCGCGTGTCGGAATCAGCACGCGCAAGAACCAAATGGGAAAATCGGAATGGGTCCAGAACTGCTTTGAGTGCTGGCTATTGGCGTCTGATTTCCGTTTTGGGAATCCTTTGCCGCGCGCCCCTCTCAAAACTCCGCACAAGTCCCAGAAAGCGGGAAAGAAATAAAACGCCACCAAAAAAAAAAAAATAAAAGCCAATCCTCGAAGCGTGGGTGGTAGGCCCTGGATTATCCCGTACAAGTATTTCTCAGGAGTAAAAAAACCGTTTGTTTTGGAATTCCCCATTTCGCGGCCACCTACGCCGCTATCTTTGCAACAACTATCTGCGATAACTCAGCAAATTTTGCATATTCGTGTTGCAGTATTGCGATAATGGGAGTCTTACTTCCAACATAACGGCAGAAAGAAATGTGAGAAAATTTTGCATCCTTTGCCTCCGTTCAAGTATATAAAGTCGGCATGCTTGATAATCTTTCTTTCCATCCTACATTGTTCTAATTATTCTTATTCTCCTTTATTCTTTCCTAACATACCAAGAAATTAATCTTCTGTCATTCGCTTAAACACTATATCAATA |
| P_HHF1_ | AAAGAAAGAATATAAAAGGTTGAGGAAAGAGATGTATCCCGAAGAATACACAGTCTTTTATATATGTATTTCAACAAGGAGCCGTGGAGGGTACCAAAAAGAAAAATCGCCCGGGCATTTCGTTATCTTCCACGCTAAAAGTCAAGGAGAGATATTACGGCCAGGATCGCAAAGGTGCAGAGCAAGGAAATGTGAGAAATTGTGAGAACGATAATGTATGGGACAATGCGAAAATGTGAGAACGAGAGCAAAAATCTTTTTTGTATCTCCCCGCCGAATTTGGAAACCGCGTTCTGAAAACTTCGCATCTTCACATAGTAAAACTGTTCCGAGCGCTTCTCCCCATAATGGTTAGTGGTAAAAACCGAAGTTGTTTACTTTAGCAAATGCCCGCGAATACGGTGGTAAATTGCCACCCCCCCTTCCCCATTCATTGGGTAAAGACCAATTTGATGGATAAATTGGTTGTGGAAAAGGTCTAATTCTTTTTCCTATAAATACCGAGATATTTTTTCTATATGATGGTTTCCGTCGCATTATTGTACTCTATAGTACTAAAGCAACAAACAAAAACAAGCAACAAATATAATATAGTAAAAT |
| P_UTR2_ | TCCGTAGTCAAGGGTTCATAATGGATGATGAGCCTGTAAACAGCTTTGTATTTACCTCTTTTACTTCAAGAATGCTCTTGCGTCGTTTGCAGTGTCGAAGAAGATCCAGTAGAGCTATACAAAATGGAGAAATGAAACAGTTGAACCAGAGCGCCGCATTTGGCAGGTAGTTTCGCTAAATTTCCTCATGCGCGTTTGTAATTATGCTAGTTAACCATATGATAAGGTTGCCTAACTTGCAGTCACCGGAAGGTTATTTCCGGAATGGTGCAGAATAATTTTTAAAATAGTAGCATATATTTCGTTCCAATTCCCAAATAGGGAATTATAATTATGACAGATAAATTCCCGATTAGGAAGAAGCGCGAAAATTCTTGAAAAACTTTTTTCAACCTCGAAGAAAATCGTCGTCCTTCGAAGATTTAGTGGGAATCTTCAAGGTGGATAGTTTCCAAATTAAAACTGCAGAAGAGGCTTTTAAAACATGCCTGAGGTATAAAAAGAACAACATTCCTGGTGATTATGGTTTCTGTTGGACCCTGTTCCCTTTGTAAAACTTCTATTTATTGTCCTGCAAATAAGCTAATTATATAATTAATA |
| P_ADH1_ | GGAGATATACAATAGAACAGATACCAGACAAGACATAATGGGCTAAACAAGACTACACCAATTACACTGCCTCATTGATGGTGGTACATAACGAACTAATACTGTAGCCCTAGACTTGATAGCCATCATCATATCGAAGTTTCACTACCCTTTTTCCATTTGCCATCTATTGAAGTAATAATAGGCGCATGCAACTTCTTTTCTTTTTTTTTCTTTTCTCTCTCCCCCGTTGTTGTCTCACCATATCCGCAATGACAAAAAAATGATGGAAGACACTAAAGGAAAAAATTAACGACAAAGACAGCACCAACAGATGTCGTTGTTCCAGAGCTGATGAGGGGTATCTCGAAGCACACGAAACTTTTTCCTTCCTTCATTCACGCACACTACTCTCTAATGAGCAACGGTATACGGCCTTCCTTCCAGTTACTTGAATTTGAAATAAAAAAAAGTTTGCTGTCTTGCTATCAAGTATAAATAGACCTGCAATTATTAATCTTTTGTTTCCTCGTCATTGTTCTCGTTCCCTTTCTTCCTTGTTTCTTTTTCTGCACAATATTTCAAGCTATACCAAGCATACAATCAACTATCTCATATACA |
| P_GLO1_ | TAAGGCAAAGTTATCACGTGAATATACTGTTTAGTATACAAGTACGTTGATTTAACGGCTGAGAGATTCTCAAACAAAGTAACGCTTAGTTTAGCTAAAGTAGAATCGAAGCGCAGTGAAGGCAAGACGAGTTATCCCTTTATCACAAAATATAAAAAATAGGTAAAGAGGGGGGTGGGGGTGGTTCGGACTTTGCATCTCGGTGCATTAGTACCACTCAGCTTCAGTTATAGGAATACTGACAATGTTCTTGAGCCAGGGAGAACTGAAATTTCCATACCCTTTAACCCTTTATTCTTGAGCATATGATCTTATTTTTCCACTCAGCACAAAATACACATCCCGCATATGATCTGGACACTGAATAAACAAGGGGCTTTACGATGGAGTAGTAGACCTGGATACGTAGTCACCACAGGGATCCTAAACTGCGTCATAGTAAGTTTCTTTGATACTAGAATGTCCCTCTCTCGTAGTAGTTGATATCCCGTATTTCTAGTTAGTGACGTTTATAAATAGGGAGAAAAAAAATCGGAATAACATTTCCATGCCTTTTTTTTGACACTTCAGACCAGATACGCCACCAGCTACAAACTAACA |
| P_POX1_ | CTCAACTCTGATCCGGATTGTCGAGGTTTCAATAAGTTACTCTGAACAACTAATCAAAATATCTCCTTATTTCTGTAGATTCCTTCAGTTCCACTTTTTACTTTTCTTAATTCTCTTTGTATTTATTCCTAGCGACGAAAAATGCGAGATCTCGACCAAAAAAAGGGGGTAGGGTAATAAAATTAACCCTATTATTTTTTAACTTTAAAACCTATAATGTGCTAATATTTTATTATAAACCTCCTTTTTTTGCGTTCAAACCCTGACACATTTTAAGCCCTATATTTACGGTATTAGTTGATTAAACTCCGAAGCGAAAGGAATTCGGTCATTAGCGGCTAATAGCCGTTGGGGTAAATCACCTACAAGCAAGTACACAAGAGAACGTTGGCGTTGTTAAGTCAAAGCACTAATACATTGGGGCTTTAAGAGTGTTTATAAAGGTCTAACCTGTAAAAATTATTTAAACAACTTGAACAGGCCTTAAAGTTTTCCTCATTCCGCTCATCATCACTAATATTGCTCTCCGTTTTTGAATACACACTTGACACTAATAAGTATCACAGAAAAAAAGAAAATATAATAAATTAGTATTGCGAT |
| P_ZWF1_ | GCCGTCGAAAAGGATCTCGTCTCTGTTGGGAGCACCTGGTAAGTAAGGTGTAGTTTTGCACCCGTGTACATAAGCGTGAAATCACCACAAACTGTGTGTATCAAGTACATAGTGACATTTAAATAATAGCAAGAACAACAATAATAGTAGCGCTACTGGAAGCACCACGTAATAGTGGAAAAGAACTGGAAAAACCGCTATAAGATGCATACTCCGGCGGTCTTACGCGGAGATACAAGCTTCCAACGGTGCTAAAAGCCCGGTTTCGGCTCGGCCGGAGGAGGAAGAGAGACGAAAAAAAAAAAAATGACTAAAAAAAAAATGGAATATTATTAATGTGGGATTTTTGGCTCAAGGTGTGGTGGCCCCTTTTCTAAGGGTGGCGAATTCTTCAATGTACGGAAAACTCGCCAAGGCTATCCCATATATAAGCAAACTGTGGGTTCATCTATATACCGACACATAACACCTAAAGTGGCTTCCTCCTGCCCCTCTCTCCCTTTTCTCCACTCACCCCTCCTTCTCCCCCTTCCCCCTCTCCAATTGGCTGTATAGACAGAAAGAGTAAATCCAATAGAATAGAAAACCACATAAGGCAAG |
| P_STE5_ | ACGAAGTGACTGACAGAATACTGACATCAGCTGATTTCTCATAGAGCTGTTTCTCTGATAACACGTTGTTTGAACATCGACAAGATGAAAATCTAGAAGTATCAAGTTTCCTTTAAAGGGATATATAACAGATTCTAAAACTGACAGAAATATTTCGAGTGAAGAAGAAGCGTTAAATATTGGATCTTTCCGCAGTTCTACTCTGATACATTTTTGAAGTAGGAGAGTCATTTAGAAGGCGTATTGCTCAATAGTAGAAAGCAGGCCTGTGCACATGAATTAATTAAAAAATATAAAGGTAGTGATTAGACGACACATGTCCATAGGTAACCTGTCATAATTTTGAACAATTTCCCTTCTTTTCTTTTTTTTTTTTGGGTGCGGCGATATGTAGCTTGTTAATTTACACATCATGTACTTTTCTGCATCAAAATATGAAAGGCGATAGTAGCTAAAGAAAATACCGAGAATTTCCTCGAAAAGTTGACGACAAAAGAAAGGCATAAAAAAGTAATTTGAAAATATTTTAAAACTGTTTTAACCCATCTAGCATCCGCGCTAAAAAAGGAAGATACAGGATACAGCGGAAACAACTTTTAA |
| P_TID3_ | AAAAAAGATGGGCGCTTAATTTCCACTCTTACAGATATCACTCCTTCTAATTAACTTACATCAGTAAGAAAGTAAAAAGAGCAACAATTCGCCACAAGAAGGTCTCTGTAGGGTCAATAGTAGCAGGGCTCTTGTCAGGGAAAGACTGTGAATGCAAAGGAAAGACGGTTGGAGGTCCGAAAAGCATTACTGATGTTAGGATTGAAGAGAAATTCACCGTACTATGTGCCAGATGGTTATAACTGAACATTGAAATAATATCGTGATTATGGGCTGGTTGAGAGCCCCGTTAAGTATTGTTAAAGATGTAAGCAGTCTATTGCGATTAGCGGTTAAAATACCATTTCAAGGGTTACCCATACTGAAAGTGCCAAAAGAAAAAAAGATAACGTATGGAATGATATAGTCACAAAACTAATATTTCCTAAAATAAACAAACAACACCAAAAATGGTAGAAATAATTGGAATACATTCACAGGAGAGGTAGAATCGTCCCTGTAAAAAAAATTGAAACGGAACAAGAAAAATAACTGACGTTCCTTTCTTTAAACGTTAGGGTGTACTATAATATATTCCATAAGAGATACCCGTTTCTATAA |
| P_SAC1_ | TCCTTGTCCTGTATAAACTACCGTACCGAGCCATTGGTCATCTTTAGACCACAAGCGAACTGTTCCATCCCTCGAAACACTAGCAACCTTTGAATCATCCACAGCTACCACATCCCTGACGTCCTGATCGTGCCCTTTAAGTGTTGCACTCAATTGATATCCCATACTCCAAATCTGCTGCTCTACACCTTACTATCACACATGAATATATATATATAAAATAAGCCAAGACAGTGGCCTTCCCTTATTATCAGCGTACTAAAATCTCATATGATTTATTTTTCGTGGTCCTGAACGAGTGTGAAAAATTTTGAAAAACTCGCAAAGGAAATGCCAAGGTCAGCAACTTTCTCGACGTCCAAGCCTCGCTCCTATTGTTTTAGTAACCTGTAGACCTTCAATATTCAAGAGCTTGCACACGCACGCATTGCTGCACTACTGCTTACCCACACTACAAATTTAATTCCAGCTTATTGAAGTTGAAAAGGCAAGGGAAAAATACCACAGGTTTAGATAAGGAAATAGGAGAAAGGATTAGAAACCATATCCCTATAACAGTAACGATAATATTTATATACACGTATATTTTCTCGTCTAGAT |

Table S2 Primers used in this study.

| Primer | Sequences (5’-3’) |
| --- | --- |
| eGFP-F | ATGGGTAAGGGAGAAGAACTTTTC |
| POT2-R | AGCCAGGTAGAGACCGAGTCACTG |
| POT2-F | CGAATTTCTTATGATTTATG |
| U1-cP_GAL1_-F | GTGACTCGGTCTCTACCTGGCTCGGATTAGAAGCCGCCGAACAATAAAGATTCTACAATACTAGC |
| U1-cP_GAL1_-R | GTTCTTCTCCCTTACCCATCCTCCTTGACGTTAAAGTATAGAGG |
| U2-cP_GAL1_-F | GACTCGGTCTCTACCTGGCTCGGGCGACAGCCCTCCGAACAATAAAGATTCTACAATAC |
| U2-cP_GAL1_-R | GTTCTTCTCCCTTACCCATCCTCCTTGACGTTAAAGTATAGAGG |
| U3-cP_GAL1_-F | GACTCGGTCTCTACCTGGCTCGGAAGACTCTCCTCCGAACAATAAAGATTCTACAATAC |
| U3-cP_GAL1_-R | GTTCTTCTCCCTTACCCATCCTCCTTGACGTTAAAGTATAGAGG |
| U4-cP_GAL1_-F | GACTCGGTCTCTACCTGGCTCGCGCCGCACTGCTCCGAACAATAAAGATTCTACAATAC |
| U4-cP_GAL1_-R | GTTCTTCTCCCTTACCCATCCTCCTTGACGTTAAAGTATAGAGG |
| U4g-cP_GAL1_-F | GACTCGGTCTCTACCTGGCTCGGGCCGCACTGCTCCGAACAATAAAGATTCTACAATAC |
| U4g-cP_GAL1_-R | GTTCTTCTCCCTTACCCATCCTCCTTGACGTTAAAGTATAGAGG |
| U5-cP_GAL1_-F | GACTCGGTCTCTACCTGGCTCGGAAAAGCGTCTTCCGAACAATAAAGATTCTACAATAC |
| U5-cP_GAL1_-R | GTTCTTCTCCCTTACCCATCCTCCTTGACGTTAAAGTATAGAGG |
| U6-cP_GAL1_-F | GACTCGGTCTCTACCTGGCTCGGCGCTCACTCTTCCGAACAATAAAGATTCTACAATAC |
| U6-cP_GAL1_-R | GTTCTTCTCCCTTACCCATCCTCCTTGACGTTAAAGTATAGAGG |
| U7-cP_GAL1_-F | GACTCGGTCTCTACCTGGCTCGGGGTGGACCACTCCGAACAATAAAGATTCTACAATAC |
| U7-cP_GAL1_-R | GTTCTTCTCCCTTACCCATCCTCCTTGACGTTAAAGTATAGAGG |
| U8-cP_GAL1_-F | GACTCGGTCTCTACCTGGCTCGGACAACTGTTGACCGAACAATAAAGATTCTACAATAC |
| U8-cP_GAL1_-R | GTTCTTCTCCCTTACCCATCCTCCTTGACGTTAAAGTATAGAGG |
| cP_GAL1_-F1 | GTGACTCGGTCTCTACCTGGCTAACAATAAAGATTCTACAATACTAGC |
| cP_GAL1_-R1 | GTTCTTCTCCCTTACCCATCCTCCTTGACGTTAAAGTATAGAGG |
| U1-cP_CYC1_-F | AGTGACTCGGTCTCTACCTGGCTCGGATTAGAAGCCGCCGCTCGAGCAGATCCGCCAG |
| U1-cP_CYC1_-R | GAAAAGTTCTTCTCCCTTACCCATCTTAGTGTGTGTATTTGTGTTTGCG |
| U2-cP_CYC1_-F | AGTGACTCGGTCTCTACCTGGCTCGGGCGACAGCCCTCCGCTCGAGCAGATCCGCCAG |
| U2-cP_CYC1_-R | GAAAAGTTCTTCTCCCTTACCCATCTTAGTGTGTGTATTTGTGTTTGCG |
| U3-cP_CYC1_-F | AGTGACTCGGTCTCTACCTGGCTCGGAAGACTCTCCTCCGCTCGAGCAGATCCGCCAG |
| U3-cP_CYC1_-R | GAAAAGTTCTTCTCCCTTACCCATCTTAGTGTGTGTATTTGTGTTTGCG |
| U4-cP_CYC1_-F | AGTGACTCGGTCTCTACCTGGCTCGCGCCGCACTGCTCCGCTCGAGCAGATCCGCCAG |
| U4-cP_CYC1_-R | GAAAAGTTCTTCTCCCTTACCCATCTTAGTGTGTGTATTTGTGTTTGCG |
| U4g-cP_CYC1_-F | AGTGACTCGGTCTCTACCTGGCTCGGGCCGCACTGCTCCGCTCGAGCAGATCCGCCAG |
| U4-cP_CYC1_-R | GAAAAGTTCTTCTCCCTTACCCATCTTAGTGTGTGTATTTGTGTTTGCG |
| U5-cP_CYC1_-F | AGTGACTCGGTCTCTACCTGGCTCGGAAAAGCGTCTTCCGCTCGAGCAGATCCGCCAG |
| U5-cP_CYC1_-R | GAAAAGTTCTTCTCCCTTACCCATCTTAGTGTGTGTATTTGTGTTTGCG |
| U6-cP_CYC1_-F | AGTGACTCGGTCTCTACCTGGCTCGGCGCTCACTCTTCCGCTCGAGCAGATCCGCCAG |
| U6-cP_CYC1_-R | GAAAAGTTCTTCTCCCTTACCCATCTTAGTGTGTGTATTTGTGTTTGCG |
| U7-cP_CYC1_-F | AGTGACTCGGTCTCTACCTGGCTCGGGGTGGACCACTCCGCTCGAGCAGATCCGCCAG |
| U7-cP_CYC1_-R | GAAAAGTTCTTCTCCCTTACCCATCTTAGTGTGTGTATTTGTGTTTGCG |
| U8-cP_CYC1_-F | AGTGACTCGGTCTCTACCTGGCTCGGACAACTGTTGACCGCTCGAGCAGATCCGCCAG |
| U8-cP_CYC1_-R | GAAAAGTTCTTCTCCCTTACCCATCTTAGTGTGTGTATTTGTGTTTGCG |
| cP_CYC1_-F | AGTGACTCGGTCTCTACCTGGCTCTCGAGCAGATCCGCCAGGC |
| cP_CYC1_-R | GAAAAGTTCTTCTCCCTTACCCATCTTAGTGTGTGTATTTGTGTTTGCG |
| U3m-cP_CYC1_-F | GACTCGGTCTCTACCTGGCTAGGAAGACtCtCCtCCGCTCGAGCAGATCCGCCAGGCG |
| U3m-cP_CYC1_-R | GAAAAGTTCTTCTCCCTTACCCATCTTAGTGTGTGTATTTGTGTTTGCG |
| POT2-cP_GAL1_-F | AACAATAAAGATTCTACAATACTAGC |
| POT2-cP_GAL1_-R | AGCCAGGTAGAGACCGAGTCAC |
| U4444-cP_GAL1_-F | GACTCGGTCTCTACCTGGCTCGCGCCGCACTGCTCCGAGCGCGCCGCACTGCTCCGACGCGCCGCACTGCTCCGTGCGTCCTCGTCTTCA |
| U4444-cP_GAL1_-R | ATTGTAGAATCTTTATTGTTCGGAGCAGTGCGGCGCGAGGCACATCTGCGTTTCAGGAACGCGACCGGTGAAGACGAGGACGCACGGAGC |
| U1234g-cP_GAL1_-F | GACTCGGTCTCTACCTGGCTCGGATTAGAAGCCGCCGAGCGGGCGACAGCCCTCCGACGGAAGACTCTCCTCCGTGCGTCCTCGTCTTCA |
| U1234g-cP_GAL1_-R | ATTGTAGAATCTTTATTGTTCGGAGCAGTGCGGCCCGAGGCACATCTGCGTTTCAGGAACGCGACCGGTGAAGACGAGGACGCACGGAGG |
| U2222-cP_GAL1_-F | GACTCGGTCTCTACCTGGCTCGGGCGACAGCCCTCCGAGCGGGCGACAGCCCTCCGACGGGCGACAGCCCTCCGTGCGTCCTCGTCTTCA |
| U2222-cP_GAL1_-R | ATTGTAGAATCTTTATTGTTCGGAGGGCTGTCGCCCGAGGCACATCTGCGTTTCAGGAACGCGACCGGTGAAGACGAGGACGCACGGAGG |
| U2223-cP_GAL1_-F | GACTCGGTCTCTACCTGGCTCGGGCGACAGCCCTCCGAGCGGGCGACAGCCCTCCGACGGGCGACAGCCCTCCGTGCGTCCTCGTCTTCA |
| U2223-cP_GAL1_-R | ATTGTAGAATCTTTATTGTTCGGAGGAGAGTCTTCCGAGGCACATCTGCGTTTCAGGAACGCGACCGGTGAAGACGAGGACGCACGGAG |
| U2224G-cP_GAL1_-F | GACTCGGTCTCTACCTGGCTCGGGCGACAGCCCTCCGAGCGGGCGACAGCCCTCCGACGGGCGACAGCCCTCCGTGCGTCCTCGTCTTCA |
| U2224G-cP_GAL1_-R | ATTGTAGAATCTTTATTGTTCGGAGCAGTGCGGCCCGAGGCACATCTGCGTTTCAGGAACGCGACCGGTGAAGACGAGGACGCACGGAGG |
| U2224-cP_GAL1_-F | GACTCGGTCTCTACCTGGCTCGGGCGACAGCCCTCCGAGCGGGCGACAGCCCTCCGACGGGCGACAGCCCTCCGTGCGTCCTCGTCTTCA |
| U2224-cP_GAL1_-R | ATTGTAGAATCTTTATTGTTCGGAGCAGTGCGGCGCGAGGCACATCTGCGTTTCAGGAACGCGACCGGTGAAGACGAGGACGCACGGAGG |
| U2233-cP_GAL1_-F | GACTCGGTCTCTACCTGGCTCGGGCGACAGCCCTCCGAGCGGGCGACAGCCCTCCGACGGAAGACTCTCCTCCGTGCGTCCTCGTCTTCA |
| U2233- cP_GAL1_-R | ATTGTAGAATCTTTATTGTTCGGAGGAGAGTCTTCCGAGGCACATCTGCGTTTCAGGAACGCGACCGGTGAAGACGAGGACGCACGGAGG |
| U2244-cP_GAL1_-F | GACTCGGTCTCTACCTGGCTCGGGCGACAGCCCTCCGAGCGGGCGACAGCCCTCCGACGCGCCGCACTGCTCCGTGCGTCCTCGTCTTCA |
| U2244-cP_GAL1_-R | ATTGTAGAATCTTTATTGTTCGGAGCAGTGCGGCGCGAGGCACATCTGCGTTTCAGGAACGCGACCGGTGAAGACGAGGACGCACGGAGC |
| U2333-cP_GAL1_-F | GACTCGGTCTCTACCTGGCTCGGGCGACAGCCCTCCGAGCGGAAGACTCTCCTCCGACGGAAGACTCTCCTCCGTGCGTCCTCGTCTTCA |
| U2333-cP_GAL1_-R | ATTGTAGAATCTTTATTGTTCGGAGGAGAGTCTTCCGAGGCACATCTGCGTTTCAGGAACGCGACCGGTGAAGACGAGGACGCACGGAGG |
| U2444-cP_GAL1_-F | GACTCGGTCTCTACCTGGCTCGGGCGACAGCCCTCCGAGCGCGCCGCACTGCTCCGACGCGCCGCACTGCTCCGTGCGTCCTCGTCTTCA |
| U2444-cP_GAL1_-R | ATTGTAGAATCTTTATTGTTCGGAGCAGTGCGGCGCGAGGCACATCTGCGTTTCAGGAACGCGACCGGTGAAGACGAGGACGCACGGAGC |
| U3333-cP_GAL1_-F | GACTCGGTCTCTACCTGGCTCGGAAGACTCTCCTCCGAGCGGAAGACTCTCCTCCGACGGAAGACTCTCCTCCGTGCGTCCTCGTCTTCA |
| U3333-cP_GAL1_-R | ATTGTAGAATCTTTATTGTTCGGAGGAGAGTCTTCCGAGGCACATCTGCGTTTCAGGAACGCGACCGGTGAAGACGAGGACGCACGGAGG |
| U4222-cP_GAL1_-F | GACTCGGTCTCTACCTGGCTCGCGCCGCACTGCTCCGAGCGGGCGACAGCCCTCCGACGGGCGACAGCCCTCCGTGCGTCCTCGTCTTCA |
| U4222-cP_GAL1_-R | ATTGTAGAATCTTTATTGTTCGGAGGGCTGTCGCCCGAGGCACATCTGCGTTTCAGGAACGCGACCGGTGAAGACGAGGACGCACGGAGG |
| U7234-cP_GAL1_-F | GACTCGGTCTCTACCTGGCTCGGGGTGGACCACTCCGAGCGGGCGACAGCCCTCCGACGGAAGACTCTCCTCCGTGCGTCCTCGTCTTCA |
| U7234-cP_GAL1_-R | ATTGTAGAATCTTTATTGTTCGGAGCAGTGCGGCGCGAGGCACATCTGCGTTTCAGGAACGCGACCGGTGAAGACGAGGACGCACGGAGG |
| U7234g cP_GAL1_-F | GACTCGGTCTCTACCTGGCTCGGGGTGGACCACTCCGAGCGGGCGACAGCCCTCCGACGGAAGACTCTCCTCCGTGCGTCCTCGTCTTCA |
| U7234g-cP_GAL1_-R | ATTGTAGAATCTTTATTGTTCGGAGCAGTGCGGCCCGAGGCACATCTGCGTTTCAGGAACGCGACCGGTGAAGACGAGGACGCACGGAGG |
| U7238-cP_GAL1_-F | GACTCGGTCTCTACCTGGCTCGGGGTGGACCACTCCGAGCGGGCGACAGCCCTCCGACGGAAGACTCTCCTCCGTGCGTCCTCGTCTTCA |
| U7238-cP_GAL1_-R | ATTGTAGAATCTTTATTGTTCGGTCAACAGTTGTCCGAGGCACATCTGCGTTTCAGGAACGCGACCGGTGAAGACGAGGACGCACGGAGG |
| UP-cP_GAL1_-F | GACTCGGTCTCTACCTGGCTCGCTTAACTGCTCATTGCTATATTGAAGTACGGATTAGAAGCCGCCGAGCG |
| UP-cP_GAL1_-R | AGTTCTTCTCCCTTACCCATCCTCCTTGACGTTAAAGTATAG |
| P_ADH1_-F | GACTCGGTCTCTACCTGGCTGGAGATATACAATAGAACAG |
| P_ADH1_-R | AGTTCTTCTCCCTTACCCATCTGTATATGAGATAGTTGATTG |
| P_CCW12_-F | GACTCGGTCTCTACCTGGCTGGATACTTCATGCTATTTATAG |
| P_CCW12_-R | AGTTCTTCTCCCTTACCCATCTATTGATATAGTGTTTAAGCG |
| P_CIT1_-F | GACTCGGTCTCTACCTGGCTCTAAGAAAAAAGGAGCCATC |
| P_CIT1_-R | AGTTCTTCTCCCTTACCCATCCTTCGTAAATAGTATTATATTGC |
| P_CIT3_-F | GACTCGGTCTCTACCTGGCTAAATAAGAAATTCTTTTATAAATAC |
| P_CIT3_-R | AAGTTCTTCTCCCTTACCCATCTTTGTATTCAATTATTTAGCGGCG |
| P_CYC1_-F | GACTCGGTCTCTACCTGGCTGAGCTCATTTGGCGAGCGTTGG |
| P_CYC1_-R | AGTTCTTCTCCCTTACCCATCTTAGTGTGTGTATTTGTGTTTG |
| P_GCN4_-F | GACTCGGTCTCTACCTGGCTGTACTTATCTTCTTATATAATAG |
| P_GCN4_-R | AAGTTCTTCTCCCTTACCCATCTTTATTTGTATTTAATTTATTTTC |
| P_GLG1_-F | GACTCGGTCTCTACCTGGCTATCTGATGATTCTTTGAACC |
| P_GLG1_-R | AAGTTCTTCTCCCTTACCCATCTCCCATCACACTAGTACGCG |
| P_GLO1_-F | GACTCGGTCTCTACCTGGCTTAAGGCAAAGTTATCACGTG |
| P_GLO1_-R | AAGTTCTTCTCCCTTACCCATCTGTTAGTTTGTAGCTGGTGG |
| P_HHF1_-F | GACTCGGTCTCTACCTGGCTAAAGAAAGAATATAAAAGGTTG |
| P_HHF1_-R | AAGTTCTTCTCCCTTACCCATCATTTTACTATATTATATTTG |
| P_PGK1_-F | GACTCGGTCTCTACCTGGCTGGGCCAGAAAAAGGAAGTGTTTC |
| P_PGK1_-R | AAGTTCTTCTCCCTTACCCATCTGTTTTATATTTGTTGTAAAAAG |
| P_POX1_-F | GACTCGGTCTCTACCTGGCTCTCAACTCTGATCCGGATTG |
| P_POX1_-R | GAAAAGTTCTTCTCCCTTACCCATCATCGCAATACTAATTTATTATATTTTC |
| P_RPH1_-F | GACTCGGTCTCTACCTGGCTGATTTTTGCGATTCATTTTTC |
| P_RPH1_-R | AAGTTCTTCTCCCTTACCCATCGATTTCACGCAACTGAATATCC |
| P_SAC1_-F | GACTCGGTCTCTACCTGGCTTCCTTGTCCTGTATAAACTAC |
| P_SAC1_-R | AAGTTCTTCTCCCTTACCCATCATCTAGACGAGAAAATATACG |
| P_TDH3_-F | GACTCGGTCTCTACCTGGCTTCATTATCAATACTGCCATTTC |
| P_TDH3_-R | AAGTTCTTCTCCCTTACCCATCTTTGTTTGTTTATGTGTGTTTATTCG |
| P_TEF1_-F | GACTCGGTCTCTACCTGGCTGCCGTACCACTTCAAAACACCC |
| P_TEF1_-R | AAGTTCTTCTCCCTTACCCATCTTTGTAATTAAAACTTAGATTAG |
| P_TID3_-F | GACTCGGTCTCTACCTGGCTAAAAAAGATGGGCGCTTAATTTC |
| P_TID3_-R | AAGTTCTTCTCCCTTACCCATCTTATAGAAACGGGTATCTCTTATGG |
| P_TPI1_-F | GACTCGGTCTCTACCTGGCTGAGACCTAACTACATAGTG |
| P_TPI1_-R | AAGTTCTTCTCCCTTACCCATCTTTTAGTTTATGTATGTGTTTTTTG |
| P_UTR2_-F | GACTCGGTCTCTACCTGGCTTCCGTAGTCAAGGGTTCATAATG |
| P_UTR2_-R | AAGTTCTTCTCCCTTACCCATCTATTAATTATATAATTAGC |
| P_STE5_-F | GACTCGGTCTCTACCTGGCTACGAAGTGACTGACAGAATAC |
| P_STE5_-R | AAGTTCTTCTCCCTTACCCATCTTAAAAGTTGTTTCCGCTGTATC |
| P_ZWF1_-F | GACTCGGTCTCTACCTGGCTGCCGTCGAAAAGGATCTCGTC |
| P_ZWF1_-R | AAGTTCTTCTCCCTTACCCATCCTTGCCTTATGTGGTTTTC |
| UAS-F | GACTCGGTCTCTACCTGGCTCGGATTAGAAGCCGCCGAGC |
| UAS-R | CGGAGCAGTGCGGCGCGAGGCACATC |
| UAS_GAL1_-P_ADH1_-F | ATGTGCCTCGCGCCGCACTGCTCCGGGAGATATACAATAGAACAG |
| UAS_GAL1_-P_ADH1_-R | CGGAGCAGTGCGGCGCGAGGCACATC |
| UAS_GAL1_-P_CCW12_-F | CCTCGCGCCGCACTGCTCCGGGATACTTCATGCTATTTATAG |
| UAS_GAL1_-P_CCW12_-R | CGGAGCAGTGCGGCGCGAGGCACATC |
| UAS_GAL1_-P_CIT1_-F | CCTCGCGCCGCACTGCTCCGCTAAGAAAAAAGGAGCCATC |
| UAS_GAL1_-P_CIT1_-R | CGGAGCAGTGCGGCGCGAGGCACATC |
| UAS_GAL1_-P_CIT3_-F | GCCTCGCGCCGCACTGCTCCGAAATAAGAAATTCTTTTATAAATAC |
| UAS_GAL1_-P_CIT3_-R | CGGAGCAGTGCGGCGCGAGGCACATC |
| UAS_GAL1_-P_CYC1_-F | GCCTCGCGCCGCACTGCTCCGGAGCTCATTTGGCGAGCGTTG |
| UAS_GAL1_-P_CYC1_-R | CGGAGCAGTGCGGCGCGAGGCACATC |
| UAS_GAL1_-cP_CYC1_-F | GCCTCGCGCCGCACTGCTCCGCTCGAGCAGATCCGCCAGGCG |
| UAS_GAL1_-cP_CYC1_-R | CGGAGCAGTGCGGCGCGAGGCACATC |
| UAS_GAL1_-P_GCN4_-F | CCTCGCGCCGCACTGCTCCGGTACTTATCTTCTTATATAATAG |
| UAS_GAL1_-P_GCN4_-R | CGGAGCAGTGCGGCGCGAGGCACATC |
| UAS_GAL1_-P_GLG1_-F | CCTCGCGCCGCACTGCTCCGATCTGATGATTCTTTGAACCG |
| UAS_GAL1_-P_GLG1_-R | CGGAGCAGTGCGGCGCGAGGCACATC |
| UAS_GAL1_-P_GLO1_-F | CCTCGCGCCGCACTGCTCCGTAAGGCAAAGTTATCACGTG |
| UAS_GAL1_-P_GLO1_-R | CGGAGCAGTGCGGCGCGAGGCACATC |
| UAS_GAL1_-P_HHF1_-F | CCTCGCGCCGCACTGCTCCGAAAGAAAGAATATAAAAGGTTG |
| UAS_GAL1_-P_HHF1_-R | CGGAGCAGTGCGGCGCGAGGCACATC |
| UAS_GAL1_-P_PGK1_-F | CCTCGCGCCGCACTGCTCCGGGGCCAGAAAAAGGAAGTGTTTC |
| UAS_GAL1_-P_PGK1_-R | CGGAGCAGTGCGGCGCGAGGCACATC |
| UAS_GAL1_-P_POX1_-F | CCTCGCGCCGCACTGCTCCGCTCAACTCTGATCCGGATTGTC |
| UAS_GAL1_-P_POX1_-R | CGGAGCAGTGCGGCGCGAGGCACATC |
| UAS_GAL1_-P_RPH1_-F | CCTCGCGCCGCACTGCTCCGGATTTTTGCGATTCATTTTTC |
| UAS_GAL1_P_RPH1_-R | CGGAGCAGTGCGGCGCGAGGCACATC |
| UAS_GAL1_-P_SAC1_-F | CCTCGCGCCGCACTGCTCCGTCCTTGTCCTGTATAAACTACC |
| UAS_GAL1_-P_SAC1_-R | CGGAGCAGTGCGGCGCGAGGCACATC |
| UAS_GAL1_-P_TDH3_-F | GCCTCGCGCCGCACTGCTCCGTCATTATCAATACTGCCATTTC |
| UAS_GAL1_-P_TDH3_-R | CGGAGCAGTGCGGCGCGAGGCACATC |
| UAS_GAL1_-P_TEF1_-F | GCCTCGCGCCGCACTGCTCCGGCCGTACCACTTCAAAACACC |
| UAS_GAL1_-P_TEF1_-R | CGGAGCAGTGCGGCGCGAGGCACATC |
| UAS_GAL1_-P_TID3_-F | CCTCGCGCCGCACTGCTCCGAAAAAAGATGGGCGCTTAATTTCC |
| UAS_GAL1_-P_TID3_-R | CGGAGCAGTGCGGCGCGAGGCACATC |
| UAS_GAL1_-P_TPI1_-F | CCTCGCGCCGCACTGCTCCGGAGACCTAACTACATAGTG |
| UAS_GAL1_-P_TPI1_-R | CGGAGCAGTGCGGCGCGAGGCACATC |
| UAS_GAL1_-P_UTR2_-F | CCTCGCGCCGCACTGCTCCGTCCGTAGTCAAGGGTTCATAATG |
| UAS_GAL1_-P_UTR2_-R | CGGAGCAGTGCGGCGCGAGGCACATC |
| UAS_GAL1_-P_STE5_-F | CCTCGCGCCGCACTGCTCCGACGAAGTGACTGACAGAATAC |
| UAS_GAL1_-P_STE5_-R | CGGAGCAGTGCGGCGCGAGGCACATC |
| UAS_GAL1_-P_ZWF1_-F | CCTCGCGCCGCACTGCTCCGGCCGTCGAAAAGGATCTCGTC |
| UAS_GAL1_-P_ZWF1_-R | CGGAGCAGTGCGGCGCGAGGCACATC |
| U1m-F | GTCTCGGTTGGCAGTGACTCGGTCTCTACCTGGCTAGGATTAGAAGCCGCCGAGC |
| U1m-R | CTTCATAACCATAAAAGCTAGTATTGTAGAATCTTTATTGTTCGGAG |
| U2m-F | GTGACTCGGTCTCTACCTGGCTCGGATTAGAAGCCGCCGAGAGGGCGACAGCCCTCCGACG |
| U2m-R | CTTCATAACCATAAAAGCTAGTATTGTAGAATCTTTATTGTTCGGAG |
| U3m-F | CAGTGACTCGGTCTCTACCTGGCTCGGATTAGAAGCCGCCGAGCGGGCGACAGCCCTCCGAAGGAAGACTCTCCTCCGTGC |
| U3m-R | CTTCATAACCATAAAAGCTAGTATTGTAGAATCTTTATTGTTCGGAG |
| U4m-F | CAGTGACTCGGTCTCTACCTGGCTCGGATTAGAAGCCGCCGAGC |
| U4m-R | TAGTATTGTAGAATCTTTATTGTTCGGAGCAGTGCGGCGCTAGGCACATCTGCGTTTCAGG |
| 4×Um-F | CAGTGACTCGGTCTCTACCTGGCTAGGATTAGAAGCCGCCGAGAGGGCGACAGCCCTCCGAAGGAAGACTCTCCTCCGTGCGTC |
| 4×Um-R | TAGTATTGTAGAATCTTTATTGTTCGGAGCAGTGCGGCGCTAGGCACATCTGCGTTTCAGG |
| U1-rC-F | CAGTGACTCGGTCTCTACCTGGCTCGGATTAGAAGCCGCCGAGAGGGCGACAGCCCTCCGAAGGAAGACTCTCCTCCGTGCGTC |
| U1-rC-R | TAGTATTGTAGAATCTTTATTGTTCGGAGCAGTGCGGCGCTAGGCACATCTGCGTTTCAGG |
| U2-rC-F | CAGTGACTCGGTCTCTACCTGGCTAGGATTAGAAGCCGCCGAGCGGGCGACAGCCCTCCGAAGGAAGACTCTCCTCCGTGCGTC |
| U2-rC-R | TAGTATTGTAGAATCTTTATTGTTCGGAGCAGTGCGGCGCTAGGCACATCTGCGTTTCAGG |
| U3-rC-F | CAGTGACTCGGTCTCTACCTGGCTAGGATTAGAAGCCGCCGAGAGGGCGACAGCCCTCCGACGGAAGACTCTCCTCCGTGCGTC |
| U3-rC-R | TAGTATTGTAGAATCTTTATTGTTCGGAGCAGTGCGGCGCTAGGCACATCTGCGTTTCAGG |
| U4-rC-F | CAGTGACTCGGTCTCTACCTGGCTAGGATTAGAAGCCGCCGAGAGGGCGACAGCCCTCCGAAGGAAGACTCTCCTCCGTGCGTC |
| U4-rC-R | TAGTATTGTAGAATCTTTATTGTTCGGAGCAGTGCGGCGCGAGGCACATCTGCGTTTCAGG |
| POT2-BGL1-F | ATGTTGATGATAGTACAGCTTTTGG |
| POT2-BGL1-R | AGCCAGGTAGAGACCGAGTCAC |
| P_GAL1_-BGL1-F | GACTCGGTCTCTACCTGGCTCGGATTAGAAGCCGCCGAGC |
| P_GAL1_-BGL1-R | CCAAAAGCTGTACTATCATCAACATCCTCCTTGACGTTAAAGTATAGAGG |
| UAS-P_TDH3_-BGL1-F | GACTCGGTCTCTACCTGGCTCGGATTAGAAGCCGCCGAGC |
| UAS-P_TDH3_-BGL1-R | CCAAAAGCTGTACTATCATCAACATCTTTGTTTGTTTATGTGTG |
| UAS-P_TEF1_-BGL1-F | GACTCGGTCTCTACCTGGCTCGGATTAGAAGCCGCCGAGC |
| UAS-P_TEF1_-BGL1-R | AGCTGTACTATCATCAACATCTTTGTAATTAAAACTTAG |
| POT2-BGL1-sed1-F | ATGTTGATGATAGTACAGCTTTTGG |
| POT2-BGL1-sed1-R | AGCCAGGTAGAGACCGAGTCAC |
| P_GAL1_-BGL1-sed1-F | GACTCGGTCTCTACCTGGCTCGGATTAGAAGCCGCCGAGC |
| P_GAL1_-BGL1-sed1-R | CCAAAAGCTGTACTATCATCAACATCCTCCTTGACGTTAAAGTATAGAGG |
| UAS_GAL1_-P_TDH3_-BGL1-sed1-F | GACTCGGTCTCTACCTGGCTCGGATTAGAAGCCGCCGAGC |
| UAS_GAL1_-P_TDH3_-BGL1-sed1-R | CCAAAAGCTGTACTATCATCAACATCTTTGTTTGTTTATGTGTG |
| UAS_GAL1_-P_TEF1_-BGL1-sed1-F | GACTCGGTCTCTACCTGGCTCGGATTAGAAGCCGCCGAGC |
| UAS_GAL1_-P_TEF1_-BGL1-sed1-R | AGCTGTACTATCATCAACATCTTTGTAATTAAAACTTAG |
| pCUT-F | GTTTTAGAGCTAGAAATAGCAAGTTAAAATAAGGC |
| pCUT-R | AAAGTCCCATTCGCCACCCGAAGGTG |
| gRNA-GAL1-F | GGCCGGCATGGTCCCAGCCTCCTCGCTGGCGCCGGCTGGGCAACACCTTCGGGTGGCGAATGGGACTTTAACCAACTATAATTTAAGAG |
| gRNA-GAL1-R | CTCGGTGCCACTTTTTCAAGTTGATAACGGACTAGCCTTATTTTAACTTGCTATTTCTAGCTCTAAAACCTCTTAAATTATAGTTGGTT |
| HOGAL1 Up-F | CCATTCTCAATTAGCTCTACC |
| HOGAL1 Up-R | AGTAAAAAAAAGAAGTATACTACTTCAATATAGCAATGAGCAG |
| HOGAL1 Dn-F | CTGCTCATTGCTATATTGAAGTAGTATACTTCTTTTTTTTACTTTG |
| HOGAL1 Dn-R | TGATAAGACGTCAACTGCCC |
| gRNA-GAL80-F | TCCTCGCTGGCGCCGGCTGGGCAACACCTTCGGGTGGCGAATGGGACTTTGTTTATAAAAGTAACATGAT |
| gRNA-GAL80-R | GTTGATAACGGACTAGCCTTATTTTAACTTGCTATTTCTAGCTCTAAAACATCATGTTACTTTTATAAAC |
| HOGAL80-F | GCAATATAATAGTTTAATTCTAATATTAATAATATCCTATATTTTCTTCATTTACAAGCATCTTGCCCTGTGCTTG |
| HOGAL80-R | CGTTTTTATAACGTTCGCTGCACTGGGGGCCAAGCACAGGGCAAGATGCTTGTAAATGAAGAAAATATAGG |
| U1m-F | GTCTCGGTTGGCAGTGACTCGGTCTCTACCTGGCTAGGATTAGAAGCCGCCGAGC |
| U1m-R | CTTCATAACCATAAAAGCTAGTATTGTAGAATCTTTATTGTTCGGAG |
| U2m-F | GTGACTCGGTCTCTACCTGGCTCGGATTAGAAGCCGCCGAGAGGGCGACAGCCCTCCGACG |
| U2m-R | CTTCATAACCATAAAAGCTAGTATTGTAGAATCTTTATTGTTCGGAG |
| U3m-F | CAGTGACTCGGTCTCTACCTGGCTCGGATTAGAAGCCGCCGAGCGGGCGACAGCCCTCCGAAGGAAGACTCTCCTCCGTGCG |
| U3m-R | CTTCATAACCATAAAAGCTAGTATTGTAGAATCTTTATTGTTCGGAG |
| U4m-F | CAGTGACTCGGTCTCTACCTGGCTCGGATTAGAAGCCGCCGAGC |
| U4m-R | TAGTATTGTAGAATCTTTATTGTTCGGAGCAGTGCGGCGCtAGGCACATCTGCGTTTCAGG |
| 4×Um-F | GTGACTCGGTCTCTACCTGGCTAGGATTAGAAGCCGCCGAGAGGGCGACAGCCCTCCGAAGGAAGACTCTCCTCCGTGCGTC |
| 4×Um-R | TAGTATTGTAGAATCTTTATTGTTCGGAGCAGTGCGGCGCtAGGCACATCTGCGTTTCAGG |
| U1rc-F | GACTCGGTCTCTACCTGGCTCGGATTAGAAGCCGCCGAGAGGGCGACAGCCCTCCGAAGGAAGACTCTCCTCCGTGCGTC |
| U1rc-R | TAGTATTGTAGAATCTTTATTGTTCGGAGCAGTGCGGCGCtAGGCACATCTGCGTTTCAGG |
| U2rc-F | AGTGACTCGGTCTCTACCTGGCTAGGATTAGAAGCCGCCGAGCGGGCGACAGCCCTCCGAAGGAAGACTCTCCTCCGTGC |
| U2rc-R | TAGTATTGTAGAATCTTTATTGTTCGGAGCAGTGCGGCGCtAGGCACATCTGCGTTTCAGG |
| U3rc-F | GTGACTCGGTCTCTACCTGGCTAGGATTAGAAGCCGCCGAGAGGGCGACAGCCCTCCGACGGAAGACTCTCCTCCGTGCGTC |
| U3rc-R | TAGTATTGTAGAATCTTTATTGTTCGGAGCAGTGCGGCGCtAGGCACATCTGCGTTTCAGG |
| U4rc-F | GTGACTCGGTCTCTACCTGGCTAGGATTAGAAGCCGCCGAGAGGGCGACAGCCCTCCGAAGGAAGACTCTCCTCCGTGCGTC |
| U4rc-R | TAGTATTGTAGAATCTTTATTGTTCGGAGCAGTGCGGCGCGAGGCACATCTGCGTTTCAGG |

Table S3 Guide RNA and homologous fragments used in this study.

| Name | Sequences (5’-3’) |
| --- | --- |
| Δ*gal80* gRNA | GTTTATAAAAGTAACATGAT |
| Δ*gal80* homologous fragments | GCAATATAATAGTTTAATTCTAATATTAATAATATCCTATATTTTCTTCATTTACAAGCATCTTGCCCTGTGCTTGGCCCCCAGTGCAGCGAACGTTATAAAAACG |
| Δ*gal1* gRNA | AACCAACTATAATTTAAGAG |
| Δ*gal1* homologous fragments | CCATTCTCAATTAGCTCTACCACAGTGTGTGAACCAATGTATCCAGCACCACCTGTAACCAAAACAATTTTAGAAGTACTTTCACTTTGTAACTGAGCTGTCATTTATATTGAATTTTCAAAAATTCTTACTTTTTTTTTGGATGGACGCAAAGAAGTTTAATAATCATATTACATGGCATTACCACCATATACATATCCATATCTAATCTTACTTATATGTTGTGGAAATGTAAAGAGCCCCATTATCTTAGCCTAAAAAAACCTTCTCTTTGGAACTTTCAGTAATACGCTTAACTGCTCATTGCTATATTGAAGTAGTATACTTCTTTTTTTTACTTTGTTCAGAACAACTTCTCATTTTTTTCTACTCATAACTTTAGCATCACAAAATACGCAATAATAACGAGTAGTAACACTTTTATAGTTCATACATGCTTCAACTACTTAATAAATGATTGTATGATAATGTTTTCAATGTAAGAGATTTCGATTATCCACAAACTTTAAAACACAGGGACAAAATTCTTGATATGCTTTCAACCGCTGCGTTTTGGATACCTATTCTTGACATGATATGACTACCATTTTGTTATTGTACGTGGGGCAGTTGACGTCTTATCA |
